# Self-replicating hierarchical modular robotic swarms

**DOI:** 10.1038/s44172-022-00034-3

**Published:** 2022-11-22

**Authors:** Amira Abdel-Rahman, Christopher Cameron, Benjamin Jenett, Miana Smith, Neil Gershenfeld

**Affiliations:** 1 Center for Bits and Atoms, Massachusetts Institute of Technology, Cambridge, MA USA; 2 U.S. Army Research Laboratory, Aberdeen Proving Ground, MD USA

**Keywords:** Mechanical engineering, Aerospace engineering, Electrical and electronic engineering, Computer science

## Abstract

Modular robotic systems built of reconfigurable components offer an efficient and versatile alternative to traditional monolithic robots. However, as modular systems scale up, construction efficiency is compromised due to an increase in travel time and path planning complexity. Here we introduce a discrete modular material-robot system that is capable of serial, recursive (making more robots), and hierarchical (making larger robots) assembly. This is accomplished by discretizing the construction into a feedstock of simple primitive building blocks which can be re-configured to create a wide range of functionality. The discretization significantly simplifies the swarm’s navigation, error correction, and coordination. The component composition is supported by an algorithm to compile the building blocks into swarms and plan the optimal construction path. Our approach challenges the convention that larger constructions need larger machines to build them, and could be applied in areas that today either require substantial capital investments for fixed infrastructure or are altogether unfeasible.

## Abstract (web summary)

Abdel-Rahman and colleagues introduce a discrete modular material-robot system that is capable of serial, recursive (making more robots), and hierarchical (making larger robots) assembly. This is accomplished by discretizing the construction into a feedstock of simple primitive building blocks combined with an algorithm to plan the optimal construction path and assemble the building blocks into functional units and swarms.

## Introduction

Construction applications are an important motivation for the development of robotic swarms, however, the practice of large-scale automated fabrication has largely focused on the use of gantries, which are limited by costs that can scale super-linearly in the machine size^1^, while offering poorer performance relative to their smaller counterparts^2^.

Biological growth spans many more orders of magnitude than a gantry, from ribosomes assembling amino acids into proteins, up to organelles, cells, organs, organisms, and populations. This dynamic range is attributable to two capabilities. First, self-replication—ribosomes can recursively make more ribosomes^3^, and second, hierarchy—at each scale, smaller building blocks are used to assemble larger constructs which in turn are used as the building blocks for the next level up^4^. Both offer exponential speed-ups over a serial process. The goal of this work is to apply these insights to robotic assembly and investigate how swarms can construct recursively and hierarchically, in order to mimic nature, where the assembly throughput increases with scaling.

Prior work has demonstrated thousand robot swarms for coordinated shape generation^5^ and package sorting and delivery for online retail^6^. Swarm systems have also been developed for fabrication and construction. Teams of wheeled robot arms can collectively 3D print a single object larger than the workspace for any individual stationary robot^7^. Other more customized robots can extrude a composite tube adjacent to teammates, resulting in a dynamic, multi-agent system^8^. Termite-inspired, wheeled robots can locomote on, build, and reconfigure 2.5D brick-based structures^9^. These examples do offer parallelization, but not hierarchy or self-replication.

The motivation behind modular robotic systems is to achieve versatility, robustness, and efficiency through composition of basic part types^10,11^. Self-reconfiguration has been demonstrated with actuated modules capable of detaching and moving relative to their neighbors then reattaching to form a newly configured robot^12^. Self-reproduction has been demonstrated^13^, but the complexity of a single module can be prohibitive to scaling and implementation. Separate actuation, structure, and energy modules have been demonstrated experimentally with customized motor and electronics systems housed within modular capsules^14^. All of these examples have been limited to laboratory demonstrations; for scalability, we consider here combined material-robot systems, where the robots and the material they build with are designed simultaneously to be indistinguishable.

Our approach is based on the reversible assembly of discrete lattice building blocks, where global precision is determined by local geometry, errors can be incrementally detected and corrected, dissimilar materials can be joined, and parts can be disassembled for reconfiguration and re-use. This has resulted in macro-scale, ultralight lattice materials with record-setting mechanical properties^15^ and mass-producibility^16^, as well as heterogeneous mechanical^17^ and robotic properties^18^. Applications include structural systems for morphing wings^19^ and ultralight aerostructures^20^, statically reconfigurable infrastructure^21^, and designs for large-scale space structures^22^.

The modular, incremental nature of a discrete material system lends itself to automation. Custom end effectors have been used to demonstrate their automated assembly using a traditional gantry system^23^. For larger applications, mobile robotic platforms, designed to interface with the discrete lattice building blocks^24^, have been demonstrated in serial assembly of 1D, 2D, and 3D cellular structures^25^. These relative carrier robots are designed to register to the lattice, enabling large-scale construction without typical penalties for metrology systems, and teams of robots can use the lattice to coordinate their motion. Smaller scale, discrete robotic systems have been demonstrated through the addition of degrees of freedom and modular actuation components^26^. These systems offer an alternative to the traditional division between monolithic robots (which are capable but not flexible) and modular robots (which are flexible but less capable), however, their numbers and sizes have been fixed. This is a lost opportunity, as these systems have lower performance and throughput with scaling because of the increase in travel time and time wasted in collision avoidance.

In this paper, we seek to address these shortcomings through the introduction of a discrete material-robot system that is capable of serial, recursive (making more robots), and hierarchical (making larger robots) assembly. We first implement a comparative scaling study of different assembly approaches and compare it to self-assembly throughput in biological systems. We then introduce a hardware system with the required capabilities. Finally, we built upon the algorithm introduced in^25^ for serial and swarm assembly and we present a new scalable algorithmic approach for robotic self-replication, shape discretization, path planning and task allocation for recursive and hierarchical assembly.

## Results

### Scaling robotic construction

**Fig. 1:**
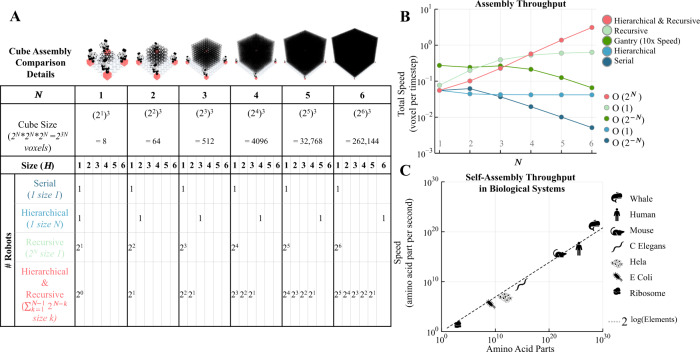
Comparative Analysis of Different Robotic Assembly Strategies vs Nature. **A** Recursive and hierarchical construction study for building a cubes of side length 2^*N*^ with size *N* = 1, 2, 3, 5, 6. **B** Assembly throughput scaling trend of different swarm configurations. **C** Throughput estimates of ribosomal assembly in biological systems^27^.

In order to evaluate the scaling benefit of recursive and hierarchical swarm assembly strategies in comparison to traditional serial monolithic construction techniques, we will consider the task of assembling a cube of side length 2^*N*^ voxels of size *d*(*N* = 1, 2, 3, 4, 5, 6) from a material pickup station with an automatic voxel feeder located at *x* = *y* = *z* = 0 (Figure 1 and detailed derivation in Fig. S4 and its following detailed description in the Supplementary Note 4).

We will start by a base case, a gantry system (similar to ref. ^23^); that assembles the voxels layer by layer and travels with the speed of *S* ⋅ *d* per second. This gantry will take ${t}_{x,y,z}=\frac{2d}{S}(max(x,y)+z)$ seconds to place a voxel at location *x*, *y*, *z* (in order to avoid collision, we assume the gantry will move in the z direction first then move in the *x* and *y* directions simultaneously); and the total construction time for the cube will be $\mathop{\sum }\nolimits_{z = 1}^{{2}^{N}}\mathop{\sum }\nolimits_{y = 1}^{{2}^{N}}\mathop{\sum }\nolimits_{x = 1}^{{2}^{N}}{t}_{x,y,z}$ s. A much simpler carrier robot (similar to ref. ^25^), will move much slower (by a factor of *F* ≤ 1) than the gantry system, and will only travel in orthogonal directions, therefore it will take ${t}_{x,y,z}=\frac{2dF}{S}(x+y+z)$ to place one voxel and the total construction time will be $\mathop{\sum }\nolimits_{z = 1}^{{2}^{N}}\mathop{\sum }\nolimits_{y = 1}^{{2}^{N}}\mathop{\sum }\nolimits_{x = 1}^{{2}^{N}}{t}_{x,y,z}=\frac{3dF}{S}({2}^{4N}+{2}^{3N})$ s. Even though the gantry system is much faster than the carrier robot, the construction time for both systems is still *O*(2^4*N*^). Moreover, as *N* increases, the cost of installing a gantry system that is bigger than the construction site increases greatly while its speed remains constant.

If we, however, introduce a carrier robot that is not only able to build the target structure, but builds identical robots from a modular material building blocks, that means it can recursively build a swarm of robot builders to parallelize the construction. If we assume the number of robots *R* is equal to (2^*N*^), and following the assumptions for the pickup stations in the supplementary section S4, *R* hence the construction time will be reduced *O*(2^3*N*^) and the robot self-assembly time is on the factor of *O*(*N*).

The introduction of self-replicating robots will first increase the assembly throughput (total voxels placed divided by total assembly time (cube and self-assembly time)) as *N* increases. However, a problem arises when *N* increases, as the carrier robots will travel a long distance from pickup stations to the target voxel placement location (specially in the *z* direction). That means that efficiency of the system and the throughput of the robots will remain constant (Fig. 1B).

Therefore, it is important to introduce modular robots that not only can build identical robots, but can also build larger robots in a hierarchical manner. These larger robots (size *H*) are able to carry hierarchical building blocks comprised of 2^3(*H*−1)^ voxels, and the small carrier robot can now assemble these blocks near the pickup station. Using this strategy, the bigger robot will take only one trip to the faraway target location instead of 2^3(*H*−1)^ trips. If there is only one hierarchical robot of size *H* = *N* to build a cube of size 2^3*N*^ voxels then the total assembly time will be *O*(2^3*N*^) and the assembly throughput will remain constant when *N* increases. If we however apply both recursive and hierarchical assembly strategies, a mix of carrier robots of different sizes having the same number of robotic parts as the hierarchical (see Fig. S4 B for details), the total assembly time is reduced to be *O*(2^2*N*^) and the assembly throughput is on the order *O*(2^*N*^).

Comparing the assembly throughput of the different robotic assembly strategies to self-assembly throughput estimates of ribosomal assembly in biological systems (Figure 1(C)^27^) we will find that the self-assembly throughput increases with the number of elements (*E*), and is of order *O*(2^*log*(*E*)^). Only by applying self-replication and hierarchy in our robotic construction were we able to reach a similar trend, in comparison to serial assembly where throughput decreased with the number of elements. This proves that self-replication and hierarchy in robotic construction should be integral factors in developing the next generation of robots for large-scale manufacturing.

### Material-robot system

**Fig. 2:**
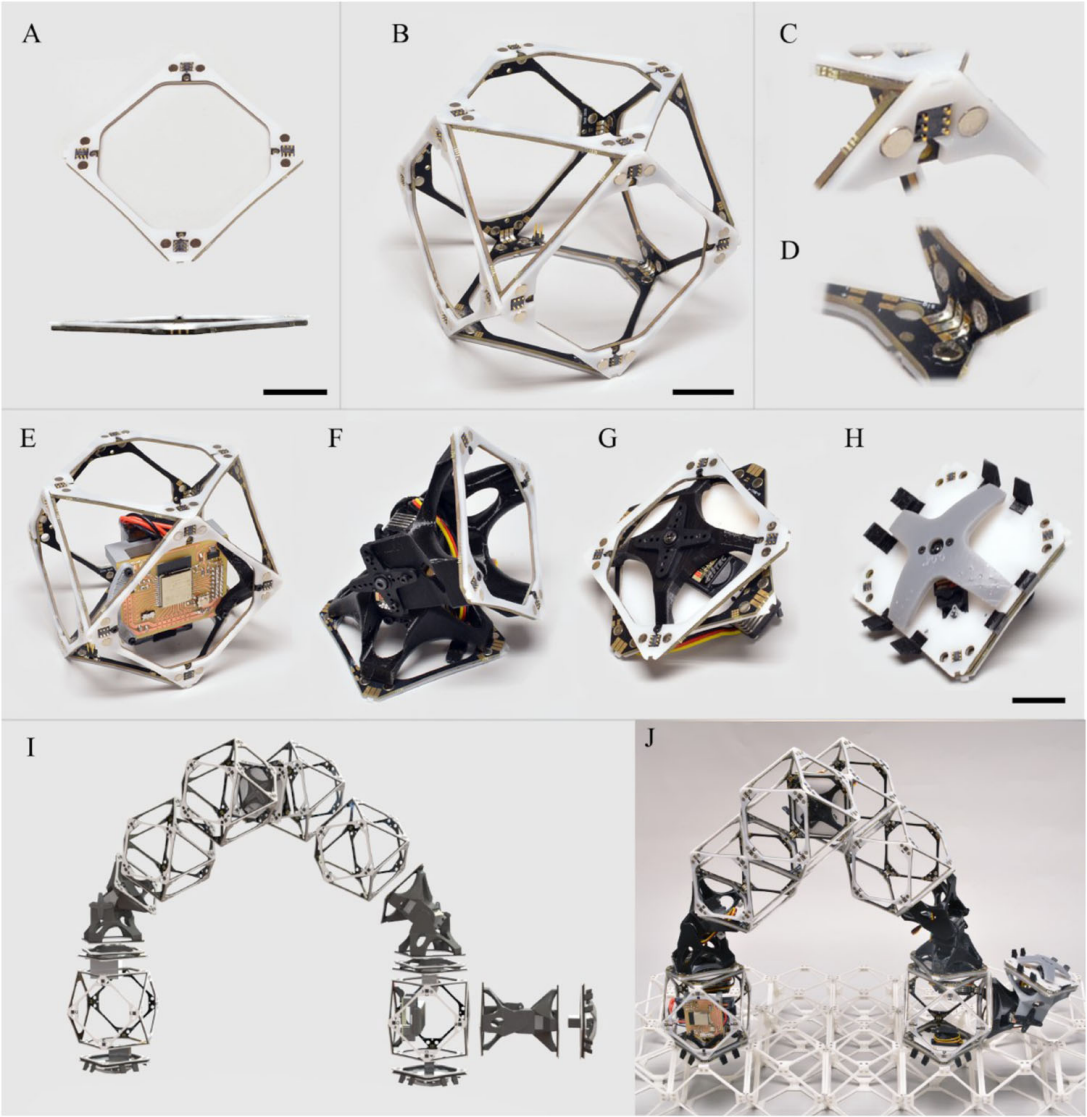
Modular building blocks and assembler. **A** Voxel face consisting of laminated PCB and acetal overlay. **B** Assembled voxel. **C** Detail view of hermaphroditic magnetic and electrical connections. **D** Detail view of internal voxel electrical connections. **E** Control and power voxel. **F** Elbow servo-actuated joint. **G** Wrist servo-actuated joint. **H** Servo-actuated gripper, **I** Exploded view rendering of assembler robot, **J** Photograph of assembler robot on passive lattice (25 mm scale bars).

The hardware developed for this paper is an extension of the system presented by Jenett et al.^25^. This previous system consisted of passive structural lattice voxels which form a substrate for the locomotion of purpose-built inchworm robots capable of rearranging and placing additional voxels. The combination of voxels and robots forms a material-robot system, enabling precise assembly of large structures with simple robots through localized registration to the underlying lattice. Our system, photographed in Fig. 2, tightens the connection of the material-robot system by creating a modular robotic toolkit that utilizes active lattice voxels as the primary structural building blocks. Combining these active voxels with actuators, control, and power enables unique behaviors including robotic self-replication and hierarchical robot assembly. A single robot, tasked with constructing a large structure, can create a heterogeneous swarm of constructors tailored to maximize material throughput for a given target geometry.

The active voxels which form the basis of the structural-robotic system are a cuboctahedral (Cuboct) unit cell which offers superior structural properties such as high stiffness at low density^17^ with geometric characteristics suited for robotic assembly and fabrication^25^. Specifically the flat Cuboct faces enable fully-constrained connections between single-voxel pairs with four points of contact, as well as a useful decomposition for voxel fabrication. The voxels in the current paper are constructed from six sides formed by laminating a printed circuit board (PCB) substrate with an acetal face sheet as shown in Fig. 2(A). These sides then combine to form the full unit cell in Figure 2(B) with soldered intravoxel electrical connections, Fig. 2(C) and epoxy reinforcement. The fully assembled voxel forms a 101.6 mm (4 in.) cube and weighs 65 grams, resulting in a lattice with an effective density of 0.062 g/cm^3^.

Figure 2(D) shows a detailed view of one of four corners involved in a face-to-face inter-voxel connection. A pair of 4.7625 mm × 3.175 mm (3/16 in. × 1/8 in.) magnets of opposite polarity create an orientation-independent structural connection while the 6-pin spring-connector creates a hermaphroditic interface for the three electrical circuits routed through each voxel face: power, ground and a single serial communication line. Each face-to-face connection can transmit 8 A at 10 V and 50 N of tensile force. Additional details of the complete fabrication process, from bare PCB to completed voxel are provided in Fig. S1. The structural-robotic system is then completed with additional active elements shown in Figure 2(E)–(H). Figure 2(E) shows a combined control and power voxel which incorporates an ESP32-based microcontroller with a 7.4 V lithium polymer battery pack. Two rotary actuators, the elbow with rotation parallel to the plane of attachment, and the wrist with rotation perpendicular to the attachment plane, are shown in Fig. 2(F) and (G), respectively. Finally, Fig. 2(H) shows a gripper designed to hold lattice elements for positioning, locomotion, and assembly. Each of these actuators incorporates an ATTiny412 microcontroller board for interfacing with the ESP32 serial commands. Details of the actuator assemblies are provided in Supplementary Fig. S2 and a complete bill of materials including weights and costs is provided in Table S1.

### Robot self-replication and hierarchy

The simplest carrier robot, shown in Fig. 2(I) and (J) is a discrete voxel analog of the purpose-built relative robot^25^. It uses inchworm locomotion, which registers to the underlying voxel lattice structure using two grippers for navigation. An additional elbow joint with a gripper allows the robot to manipulate voxels to reconfigure the lattice structure. This robot is capable of carrying a single voxel or actuator at a speed of 1 voxel per time step. While this design forms the basic unit of robotic assembler, the modular system and lattice compatible connections enable both the construction of additional assemblers by a base assembler, self-replication, as well as the production of larger robots, forming a hierarchy of assemblers.

**Fig. 3:**
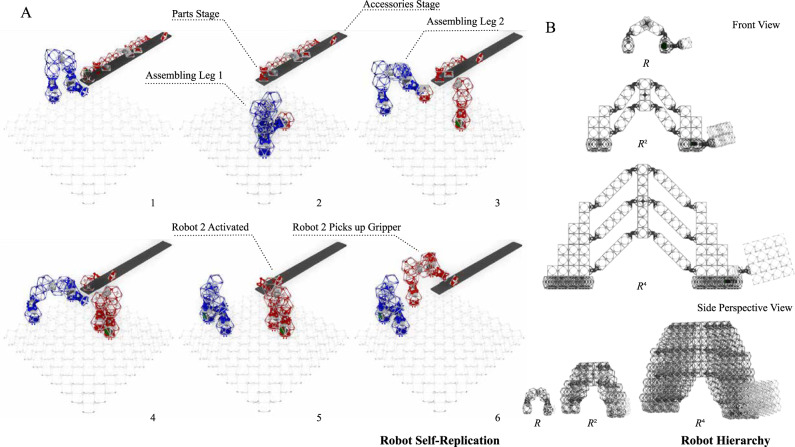
Robot self-replication and hierarchy. **A** Image stills in sequential order (1–6) of robot self-replication simulation (see Movie S2) according to the assembly sequence in Algorithm S1. **B** Proposed design for carrier robots of size *R* (*H* = 1), *R*^2^ (*H* = 2) and *R*^4^ (*H* = 3) that can carry hierarchical cubic building blocks of size 1, 2, and 4, respectively.

The algorithm for robot reproduction is constrained by two requirements. First, the new robot must be free to move after assembly. While this requirement seems trivial, it precludes building flat on the lattice substrate and subsequently lifting the completed robot into position due to the nature of the magnetic connections. To overcome this, child carrier robots are built out from a base consisting of a control voxel and gripper which is capable of anchoring to the lattice. Second, only three components of the robotic construction kit may be directly manipulated by the gripper: base voxels, control, and power voxels, and elbow joints. Both the wrist and gripper modules are designed to attach directly to a free voxel face. To accomplish this a carrier robot first picks up one of the first three-part types, then maneuvers to attach wrists and grippers to the base part before placing in the assembly in a process we call accessorizing. The grippers have electrical interfaces so that actuators, including elbows, wrists, and grippers, may be controlled by the assembler. This self-replication process is shown in Fig. 3(A) as well as Movie S2, with detailed steps outlined in Algorithm S1.

Conceptual drawings of a family of assembler robots, designed to carry voxel cubes of side lengths one two and four respectively, are shown in Figs. 3(B) and S3. The larger designs maintain the inchworm architecture while employing four-bar linkage assemblies to enable multiple actuators to act in parallel on a single degree of freedom. These early studies indicate that the number of actuators scales with a factor of 2 when *H* increases, while the total number of robotic elements scales with a factor of 2.5, lower than the geometric scaling of 3 due to tailored sparsity of the larger lattice structures. Additional gains in actuation and structural efficiency may be possible by exploring additional morphologies beyond the inchworm configuration.

### Inverse problem: from structure to swarm

Using the proposed material-robot system, we will next present algorithms that determine the optimal swarm configuration and path planning needed to build a target structure. First, we will discuss the baseline assumptions behind the path planning and simulation of the self-replicative and hierarchical swarm construction model. We are building on the assumptions made in ref. ^25^ where we assumed a centralized path planning and control strategy. The centralized control system is responsible for (1) processing and discretizing input target geometry into building blocks, (2) determining the building blocks build sequence, and (3) the task allocation and path planning based on the number of available robots. The carrier robots, on the other side, are intended to have minimal communication and computational requirements. They can only communicate with the centralized system at the pickup stations and are designed to receive and conduct one task at a time. A task entails picking up a building block from the pickup station in the form of a list of high-level step instructions towards the drop-off location (step forward, right, left, up, down, concave or convex turn)^24^, as well as the steps needed to go back to the same or a closer pickup location. The carrier robot only stores the mapping for the high-level steps to robot position, hence, memory requirement of the carrier robots depends on the size of the target build structure, i.e., based on the furthest voxel expected to be placed. In order to make the system more robust and resilient to uncertainties and disturbances, the robots could be equipped with tactile sensors that ensure that the instructed step is valid (the voxel exists and is empty) before the robot places its leg to take a step^25^. If there is a discrepancy, an emergency protocol is initiated where the robot takes the reverse steps to go back to the pickup station to notify the central system; or in case this scenario is interrupted it will stay in place and fire an emergency signal to the central system for other task-specific robots to fix the problem.

The presented algorithm assumes synchronous time stepping, where each robot spends *S* seconds (one timestep) to either take a step or pick up/drop off a voxel. For larger hierarchical robots, because of actuation limits, their speed is set to 2^*H*−1^ timesteps per step. The centralized system has a spatial-temporal Markov model of the system, where it predicts and stores the state of the system (voxel occupancy and robot locations) at each timestep in the future based only on the current state (for simplicity). For each building block in the queue to be built, the centralized system greedily finds the shortest path from the pickup station to the target drop-of location, based on an A* tree search algorithm, avoiding collision with other robots that already have tasks assigned. The carrier robots’ clocks are synced with the central system when they return to the pickup station, which also function as charging stations. Based on the expected fault tolerance *T* in the system synchronicity as well as the expected maximum steps taken, the central system adjusts the collision avoidance strategy introducing a safety factor where it makes sure, based on the spatial-temporal model, the robots do not collide within ± *T* timesteps.

The algorithm is intended to be flexible, as the number of robots in the system and pickup stations is not assumed to be fixed. During the construction, one can add/remove a robot, or a pickup station as needed, and the centralized robot will adjust its spatial-temporal model based on the new changes. Verification of the structural integrity of the lattice while being built (with imposed static and dynamic loads of robots) will be left for future work, with numerous existing precedents of solutions to this problem^23^. However, this verification will not alter the relative construction time and the speedup that recursive and hierarchical assembly offer.

### Building sequence

The centralized system’s first step is to automatically generate the build sequence given target shape. We present a scalable adaptive shape compiler that discretizes the input geometry into ordered hierarchical building blocks. The target built shape is input from the user in the form of a connected mesh, and the compiler generates its signed distance field (SDF), which represents the closest signed distance from any point in the 3D space to the surface of the mesh. We used the Gilbert-Johnson-Keerthi method^28^ to calculate the distance field using Minkowski difference for convex meshes, and the approach presented in ref. 29 for non-convex meshes. We used octree space subdivision to calculate the adaptively sampled distance field (ASDF)^30^, which subdivides the mesh into variably sized building blocks, that are larger at the center of the mesh and smaller near the surface. Minimum and maximum building blocks sizes are prescribed based on the target build resolution and maximum desired carrier robot size.

**Fig. 4:**
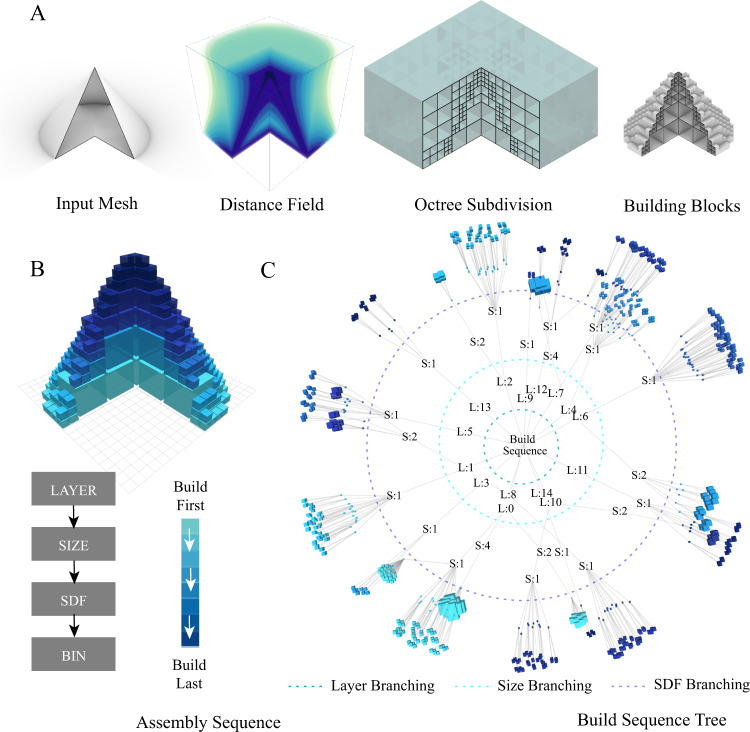
Adaptive shape compiler. **A** Hierarchical shape discretization steps using adaptively sampled distance fields (ASDF). The distance field is colored based on the distance to the input mesh surface. **B** The hierarchical building blocks are colored according to the assembly sequence. The blocks are ordered into bins first according to their layer number (*L*0 to *L*14), then their size (*S*1, *S*2, and *S*4), and finally their signed distance function (SDF) value, **C** Visualization of the final build sequence tree, all blocks that belong to the same bin can be built in parallel.

The next step is to determine the optimum sequence to assemble the building blocks, while avoiding any deadlocks in the construction. The target is to divide the building blocks into bins, whereas all blocks that belong to the same bin can be built in parallel (see Fig. 4). Since all the pickup stations are assumed to be located at the ground plane of the construction site, the structure is set to be built layer by layer, from bottom to top, which restricts the family of structures that can be built to ones that do not have underhangs. Before starting the construction, the robots build a substrate layer with an offset around the built area. Next, for each layer, the larger building blocks are assembled first since they inherently have higher SDF values. The building blocks with the same size are then grouped into bins according to their centers’ SDF value (with a tolerance that is proportional to the building block size, see Fig. 4(B)). Figure 4(C) visualizes the build sequence tree for an example cone mesh (radius 10 voxels and height 16 voxels).

### Swarm configuration and task allocation

**Fig. 5:**
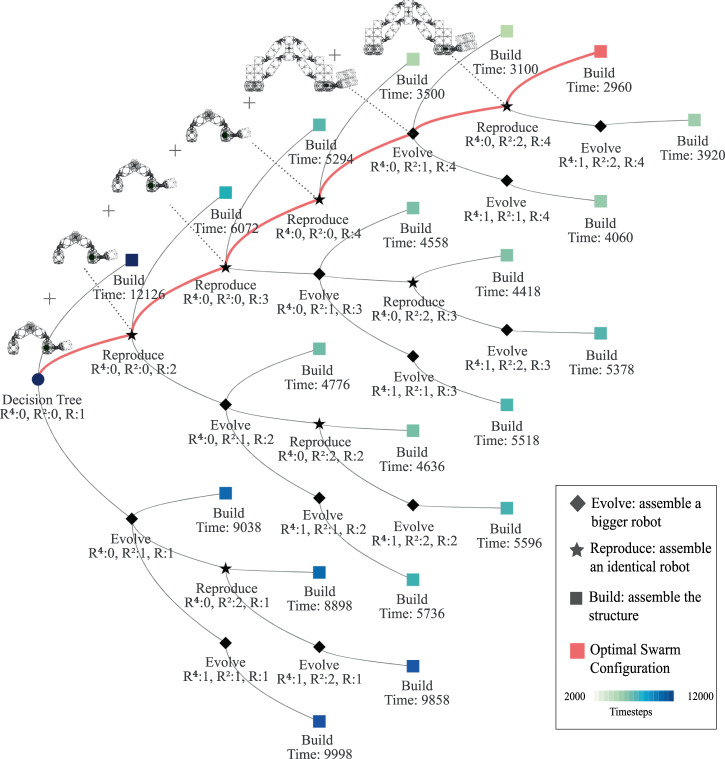
Robot decision tree. To determine the optimum swarm configuration (number of robots and their sizes), each robot can either build an identical robot (reproduce), a bigger robot (evolve) or start construction (build). We then traverse the tree searching for the optimal configuration (smallest build time given a maximum number of robots). This is the tree generated to build the example cone in Fig. 4(B), with a maximum of 4, 2, 1 robot of sizes *R* (*H* = 1), *R*^2^ (*H* = 2), and *R*^4^ (*H* = 3) respectively.

Now that the adaptive shape compiler generated the build sequence tree, the central control system can search for the optimal swarm configuration: the number of carrier robots in each size that is able to build the structure in a minimum number of timesteps. Inspired by a biological analogy, where a carrier robot has the option to either to build the structure (build), build another robot (reproduce) or build a bigger robot (evolve), we generate the tree of all possible alternatives, given a finite amount of robotic building blocks stock (see Fig. 5). Next, we traverse the tree searching for the configuration that will take the minimum amount of timesteps to be built (time to build both the robots and the structure). Once the optimal number of robots is calculated, the central system sends out instructions for the first carrier robot to assemble the rest of the robots in a recursive manner.

**Fig. 6:**
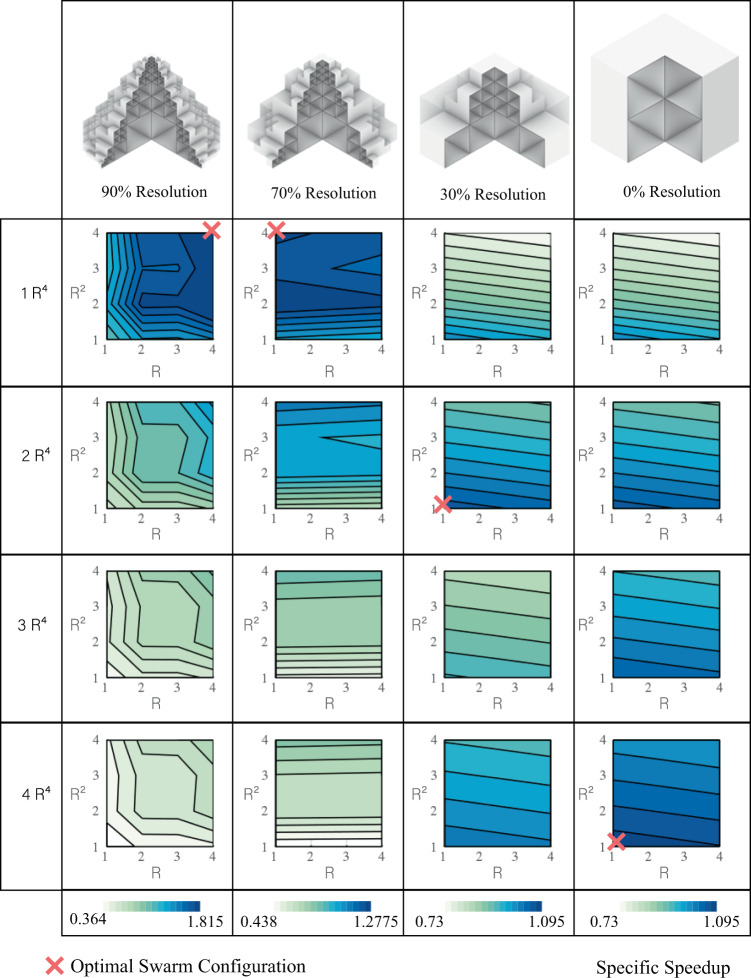
Specific speedup and optimum configuration. The specific speedup, a measure of construction efficiency, is calculated (for the target shape (cone) with different resolutions using 1, 2, 3, and 4 robots of size *R* (*H* = 1), *R*^2^ (*H* = 2), and *R*^4^ (*H* = 3)) by dividing the construction speedup (compared to construction with 1 robot of each size) by the effective number of robots (total number of robotic parts in the system). The dark blue color suggests higher specific speedup.

When going beyond homogeneous cubes as target-built structures, it is important to note that the target shape morphology and resolution greatly affect the benefit of hierarchical and recursive construction strategies. Figure 6 shows contour plots of the swarm-specific speedup for a range of heterogeneous swarms building the reference cone at four different resolutions (smoothness scale from 0 (no octree subdivisions, the final voxelized structure is just the bounding cube) to 100 (very fine subdivisions where the final voxelized structure is identical to the input mesh)). We define the specific speedup as the construction speedup (compared construction with one robot of each size) divided by the effective number of robots (total number of robotic parts in the system). The swarm configurations with the highest specific speedup are the ones that are most optimal in terms of construction throughput per unit assembly robot.

At high resolutions, the most efficient swarms employ only a single *R*^4^ (*H* = 3) robot as more of the build time is consumed placing detailed features. Conversely, the swarms with the highest specific speedup for the coarse geometries consist of only a single *R* and *R*^2^ (*H* = 2) robot with multiple of the largest *R*^4^ (*H* = 3) robots. In all cases, the optimal swarms outperform a single assembler robot in throughput per assembler by between 9 and 80%. Absolute construction speedup is shown in Supplementary Fig. S5 where we see that larger swarms always build the target structure faster, though at the cost of decreased efficiency in terms of speedup per robot.

**Fig. 7:**
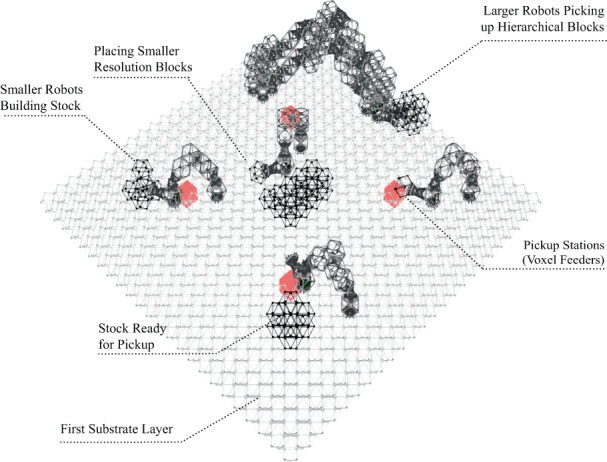
Hierarchical construction. Example construction site where smaller robots build hierarchical building blocks near the pickup locations for the bigger robots to pick up and place them in the final destination.

Finally, now that the robots are built, the construction of the target structure begins. A pseudo-code for the task allocation and planning strategy is presented in Algorithm S2. The assembly happens in a hierarchical manner, where smaller robots build stock in situ (beside the pickup station) for the bigger robots to place in their final destination (see Fig. 7). In order to test the presented algorithm, we have developed a simulation environment (details in *Materials and Methods* section). A full simulation of the swarm construction of the cone illustrated in Fig. 4(B) can be seen in the Supplementary Movie S3.

## Discussion

We have introduced a material-robot system that extends the concept of robotic swarms by being able to construct serially, recursively (making more robots), and hierarchically (making larger robots). This freedom in component composition is supported by algorithms for compiling shapes into swarms for constructing them and for planning their paths, and we evaluated their scaling through simulation showing the geometry dependence of these heterogeneous swarms. The hardware presented is an instantiation of a collective robotic system capable of recursive and hierarchical construction. It is used as an exemplary embodiment to do the scaling calculations/analysis presented, and it serves as a guideline for the requirements needed for future development. Future work will combine these contributions at scale. The system shown uses magnetic connections between the voxels. These are sufficient to demonstrate robotic assembly and locomotion, but joints with improved stiffness and strength will be required for continuum metamaterial behavior in higher-performance structures^17^. For ease of automation recent work has demonstrated that this can be achieved with a captive, interlocking, androgynous fastener^31^, and other possible solutions include electropermanent magnets^32^ and bistable latches^33^.

For actuation, we used commercially available digital servo motors, with an average net force output of roughly 57 N/kg and upper torque limits of around 10 N m^34^. This granularity limits the system scalability, which could be addressed through the serial and parallel addition of one-bit actuators^26^. Beyond self-replication and hierarchy, this system also enables a large design space in robot morphology. Preliminary work has shown a range of kinds of locomotion through distributed deformations rather than rigid joints. Robot design tools, such as those for 3D printed robotic creatures^35,36^, will be needed to simulate and optimize over these expanded degrees of freedom.

The algorithms presented are centralized; scalable compilers^37^ and decentralized control strategies^38^ will be needed as the system size grows. These algorithms provided constructive demonstrations but are not shown to be optimal; more complex path planning and collision avoidance strategies could be implemented to decrease the construction time^39^, and exploring the number and location of pickup stations is an important design parameter that greatly affects the robots’ throughput^25^. Finally, simulation to determine stress and deflection of the structure being built under static and dynamic robot loading can be pre-computed or monitored in real time for larger structures^40^.

These swarms have a range of potential applications in areas that today either require substantial capital investments for fixed infrastructure or are infeasible; candidates include seismic metamaterials^41^, automobile assembly lines^42^, aircraft subsystems^43^, and airframes^44^.

The introduction of self-replication and hierarchy into robotic swarms challenges the historical correlation between large objects needing large machines to make them. The ability of this system to span both scale and number offers the potential for exponential increase in capacity when it is needed, and, just as importantly, an exponential reduction when it is not. Sharing the same underlying principles, this promises to bring the dynamic range of biological growth into manufacturing processes.

## Methods

### Hardware fabrication and components

The material robot system employs a variety of off-the-shelf and custom hardware. A complete bill of materials is provided in Table S1. Figure S1 illustrates the assembly process for a single voxel face and a complete voxel. Voxel circuit boards were printed by PCBWay custom prototyping using 1.6 mm FR4 substrate and 1oz copper layers. Acetal faces were laser cut using a Trotec Speedy-100 Flexx and laminated to voxel circuit boards using Loctite SF-770 primer and Loctite 401 adhesive. Figure S2 shows disassembled actuator assemblies. The elbow and wrist actuators use Hitec D950TW servo motors while the gripper is actuated by a smaller Hitec HS-5087MH servo. Elbow and wrist components produced by fused deposition modeling of PLA using Prusa MK3 printers. The gripper rotary actuator was 3D printed using stereolithography of Formlabs Tough1500 resin and the Form 3 printer. The ESP32 control board and servo serial interface boards were milled on a Roland MDX-20 milling machine.

### Simulation environment

We have developed a dedicated robotic swarm simulation environment, a digital twin, to test the presented material robot system. First, we have developed an inverse kinematics model for the carrier robot to get the prescribed sequence of motor angles for each of the high-level robot steps. We then implemented the algorithms presented in the paper, in particular the self-replication demo (Algorithm S1) and hierarchical path planning and assembly (Algorithm S2, see Movie S2, S3). The simulation tool was coded in JavaScript and Three.js (https://threejs.org/) was used for 3d visualization.

### Supplementary information

Abdel-Rahman_PR File

Supplementary Material

Description of Additional Supplementary Files

Supplementary Movie 1

Supplementary Movie 2

Supplementary Movie 3

## Data Availability

Authors can confirm that all relevant data are included in the paper and/or its supplementary information files.

## Code availability

Code for the robots’ simulation can be found at: https://gitlab.cba.mit.edu/amiraa/self-replicating-hierarchical-robotic-swarms
