# Supplementary material for: Self-replicating hierarchical modular robotic swarms
Source: Commun Eng. 2022 Nov 22;1:35. doi: 10.1038/s44172-022-00034-3 (PMC10955888; doi:10.1038/s44172-022-00034-3)
Supplement: Supplementary file 2 — Supplementary Material [file 44172_2022_34_MOESM2_ESM.pdf]

Supplementary Materials

Table S1. Robot Material System Bill of Materials.

Voxel Face			
Component	*Weight [g]*	*Price [USD]*	*QTY*
Voxel PCB 1.6mm 1oz Cu	4.9	$1.89	1
Molex 788641001 Connector	0.04	$0.18	4
3/32" Diameter x 1/8" N48 Magnets	0.41	$0.12	4
Acetal Overlay, 1.6mm	4.2	$0.40	1
**Totals**	10.9	$3.49	10
**Voxel**			
Component	*Weight [g]*	*Price [USD]*	*QTY*
Voxel Face	10.9	$3.49	6
**Totals**	65.4	$20.94	6
**Elbow Actuator**			
Component	*Weight [g]*	*Price [USD]*	*QTY*
Voxel Face	10.9	$3.49	2
Hitec D950TW Servo	68	$110.49	1
ATTiny412 Servo Serial Board	2.8	$0.45	1
FDM Active Arm	23.4	$0.98	1
FDM Passive Arm	26.7	$1.12	1
Thin section bearing	3.5	$1.89	1
m3 Heat Set Insert	0.475	$0.13	2
m2 Heat Set Insert	0.18	$0.14	8
m3x5 SHCS	0.65	$0.09	2
m2x5 SHCS	0.225	$0.14	8
**Totals**	151.69	$124.55	27
**Wrist Actuator**			
Component	*Weight [g]*	*Price [USD]*	*QTY*
Voxel Face	10.9	$3.49	2
Voxel Face (Wrist)	16.3	$3.49	1
Hitec D-950TW Servo	68	$110.49	1
ATTiny412 Servo Serial Board	2.8	$0.45	1
FDM Active Arm	7	$0.15	1
m2 Heat Set Insert	0.18	$0.14	4
m2x5 SHCS	0.225	$0.14	8
**Totals**	118.42	$123.22	18
**Gripper**			
Component	*Weight [g]*	*Price [USD]*	*QTY*
Voxel Face	10.9	$3.49	1
Voxel Face (Gripper)	16.4	$3.49	1
Hitec HS-5087MH Servo	21.9	$35.27	1
ATTiny412 Servo Serial Board	2.8	$0.45	1
SLA Gripper	7.2	$1.05	1
FDM Locating Bosses	0.52	$0.02	8
m3x5 SHCS	0.65	$0.09	2
m2x5 SHCS	0.225	$0.14	2
**Totals**	65.11	$44.37	17
**Control And Power Unit**			
Component	*Weight [g]*	*Price [USD]*	*QTY*
ESP32 WROOM-32D PCB	13.9	$3.80	1
7.4V, 1 Ah Li Polymer Battery	54	$12.99	1
FDM board support	1.69	$0.04	2
FDM battery support	2.69	$0.06	2
m2 Heat Set Insert	0.18	$0.14	6
m2x5 SHCS	0.225	$0.14	6
**Totals**	79.09	$18.66	18

Algorithm S1 Self-replication Sequence

Pick up control and power voxel (c_1)
Accessorize with gripper
Anchor to substrate with gripper (g_1)
Pick up elbow actuator (e_1)
Accessorize with wrist actuator (w_1)
Attach to control and power voxel (c_1) via wrist actuator (w_1)
Pick up voxel (v_1)
Accessorize with wrist actuator (w_2)
Attach to elbow actuator (e_1) via voxel (v_1)
Pick up voxel (v_2)
Attach to wrist actuator (w_2)
Pick up voxel (v_3)
Accessorize with gripper (g_2)
Anchor to substrate with gripper (g_2)
Pick up elbow actuator (e_2)
Accessorize with wrist actuator (w_3)
Attach to voxel (v_3) via wrist actuator (w_3)
Control voxel (c_1) **moves** voxel (v_2) to **connect** to elbow actuator (e_2)
Child Bill-E now capable of lattice locomotion
Pick up elbow (e_3)
Accessorize with gripper (g_3)
Attach elbow (e_3) to voxel (v_3)

Algorithm S2 Task Allocation

for each bin B in the Building Sequence Tree **do**
while $B \subset$ building block b **do**
$S \leftarrow$size of the building block b
if there is robot same size S in the system **then**
if there is available stock same size S **then**
if robot R same size S in at any pickup station **then**
Assign R to pick-up stock and place it at target location
Remove b from bin B
end if
else
Add stock (8 size $S-1$ blocks) in front of the bin B to be built in situ
end if
else
Divide b into 8 size $S-1$ blocks & add to B to build at new target locations
Remove b from bin B
end if
end while
end for

Supplementary Note 1

Figure S2 shows each of the active modular robotic modules broken down into component pieces. Each actuator incorporates voxel faces on both sides for attaching to the lattice. One acetal overlay in the wrist actuator and gripper are modified to mount with the Hitec servos as shown in Figures S2(A) and (C). The wrist and elbow actuators use FDM printed PLA for major structural components, while the gripper rotary actuator is printed using SLA for smoother surface finish. All three actuators use ATTiny based circuit boards to interface the PWM controlled servos with the voxel serial bus.

Supplementary Note 2

To build a X by Y by Z cuboid from a pickup station that is located at $x = y = z = 0$ (see Figure S4 A1), a carrier robot that takes 1 timestep to travel 1 voxel will take a total construction time:

$$T = 2\sum_{z=1}^{Z}\sum_{y=1}^{Y}\sum_{x=1}^{X} x + y + z = XYZ^2 + YZX^2 + ZXY^2 + 3XYZ \tag{S1}$$

When $X = Y = Z = 2^N$:

$$T = 3(2^{4N} + 2^{3N}) \tag{S2}$$

If we have 2 robots, we will assume each robot will assemble a $\frac{X}{2}$ by Y by Z (see Figure S4 A2) cuboid hence

$$T = \frac{XYZ^2}{2} + \frac{XZY^2}{2} + \frac{ZYX^2}{4} + \frac{3XYZ}{2} = \frac{5}{4}\cdot 2^{4N} + \frac{3}{2}\cdot 2^{3N} \tag{S3}$$

In general for R robots, each will assemble a $\frac{2\cdot X}{R}$ by $\frac{Y}{2}$ by Z (see Figure S4 A4) cuboid, hence:

$$T = \frac{2XYZ^2}{2R} + \frac{2XZY^2}{4R} + \frac{4YZX^2}{2R^2}) + \frac{3\cdot 2XYZ}{2R} = (\frac{2}{R^2} + \frac{3}{2R})2^{4N} + \frac{3}{R}2^{3N} \tag{S4}$$

For recursive construction, $R = 2^N$, substituting in the equation S4 $T = \frac{3}{2}2^{3N} + 5\cdot 2^{2N}$. It is important to note that the maximum number of robots based on the will be $2\cdot 2^N$, hence $T = \frac{3}{4}2^{3N} + 2\cdot 2^{2N}$ (still $O(2^{3N})$).

For hierarchical construction, robot of size H contains $2\cdot 3^H$ functional voxels. If it takes 72 timesteps to build a robot of size 1 (substituting N=1 in equation S1). The self-assembly time for a robot of size H $\sum_{i=1}^{H} 2^{i-1}\cdot 2^{3(H-i)}\cdot 72$ and when it takes $\sum_{i=1}^{N} 2^{i-1}\cdot 2^{3(N-i)}\cdot 72$ timesteps to build a cube with side length 2^N. 2^{i-1} is the slow down factor, and $2^{3(N-i)}$ is the number of cubes/stock needed to build a cube of size N.

Finally for hierarchical and recursive construction the self assembly time is $\sum_{i=1}^{N-1} \frac{2^{i-1}\cdot 2^{3(N-1-i)}\cdot 12}{2^{N-1-i}}$ and construction time is $\sum_{i=1}^{N-1} 2^{i-1}\cdot 416$ (416 is the result of substituting $N = 2$ in equation S2).

Supplementary Note 3

Figure S5 shows the total construction time needed to build a cone with different resolution using a variety of swarm configurations. For the high-resolution structure, changing the number robots of size R and R^2 (self-replication) greatly alters the build time, while adding more R^4 robots (hierarchy) has minimal effect on the total build time. For the 70% resolution structure, the total construction time of the intermediate is insensitive to adding robots of size R, but decreases with additional robots of size R^2 and R^4. For the two coarsest structures, the benefit of adding R^4 robots greatly exceeds the benefit of adding additional robots of size R and R^2. Finally, even though the coarse resolution structure's volume is larger than the fine resolution one, it can be built in a faster time using the same number of robots because the robots have higher throughput, because they take leverage of hierarchical construction. This stresses on the importance of the reconfigurability of robotic construction swarms and their ability to self-assemble to optimum configurations based on the size and morphology of the target built structure.

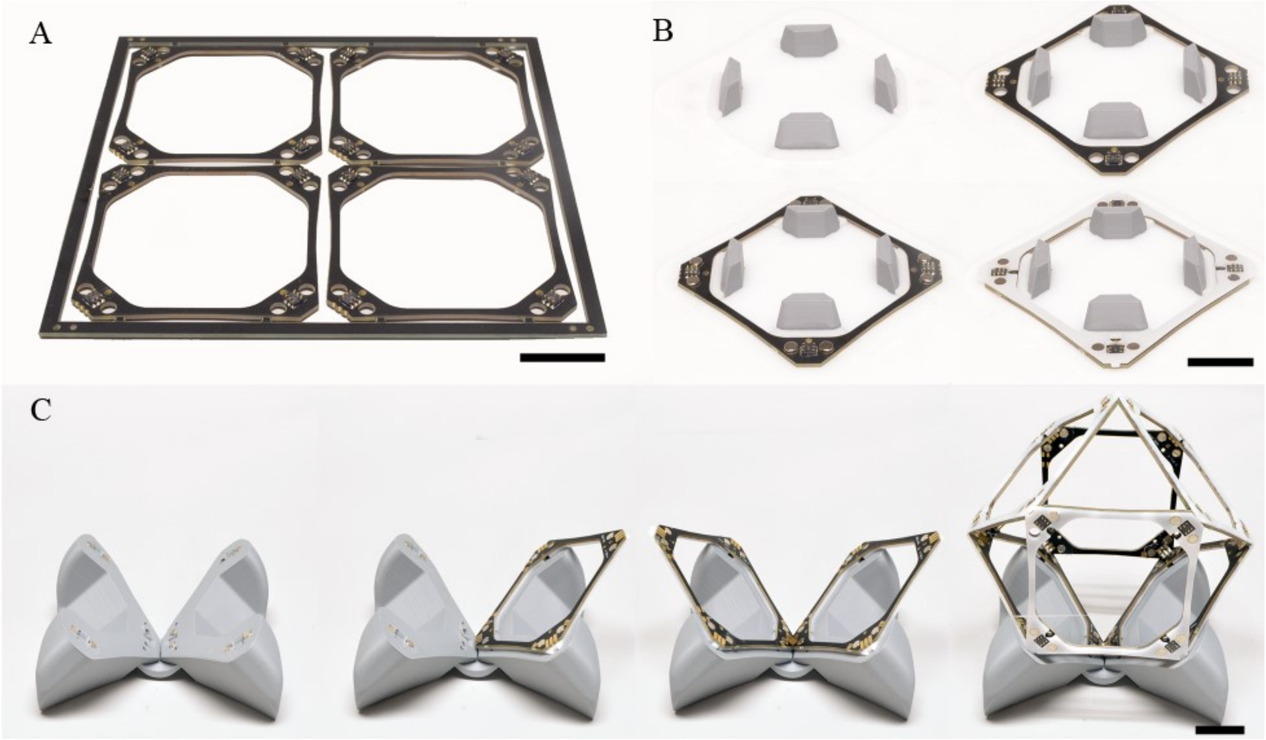

Figure S1. **Voxel manufacturing.** **(A)** Panelized circuit board after attaching smd connectors, **(B)** Tooling and lamination of voxel side, **(C)** Tooling and assembly of sides to form complete voxel (25 mm scale bars)

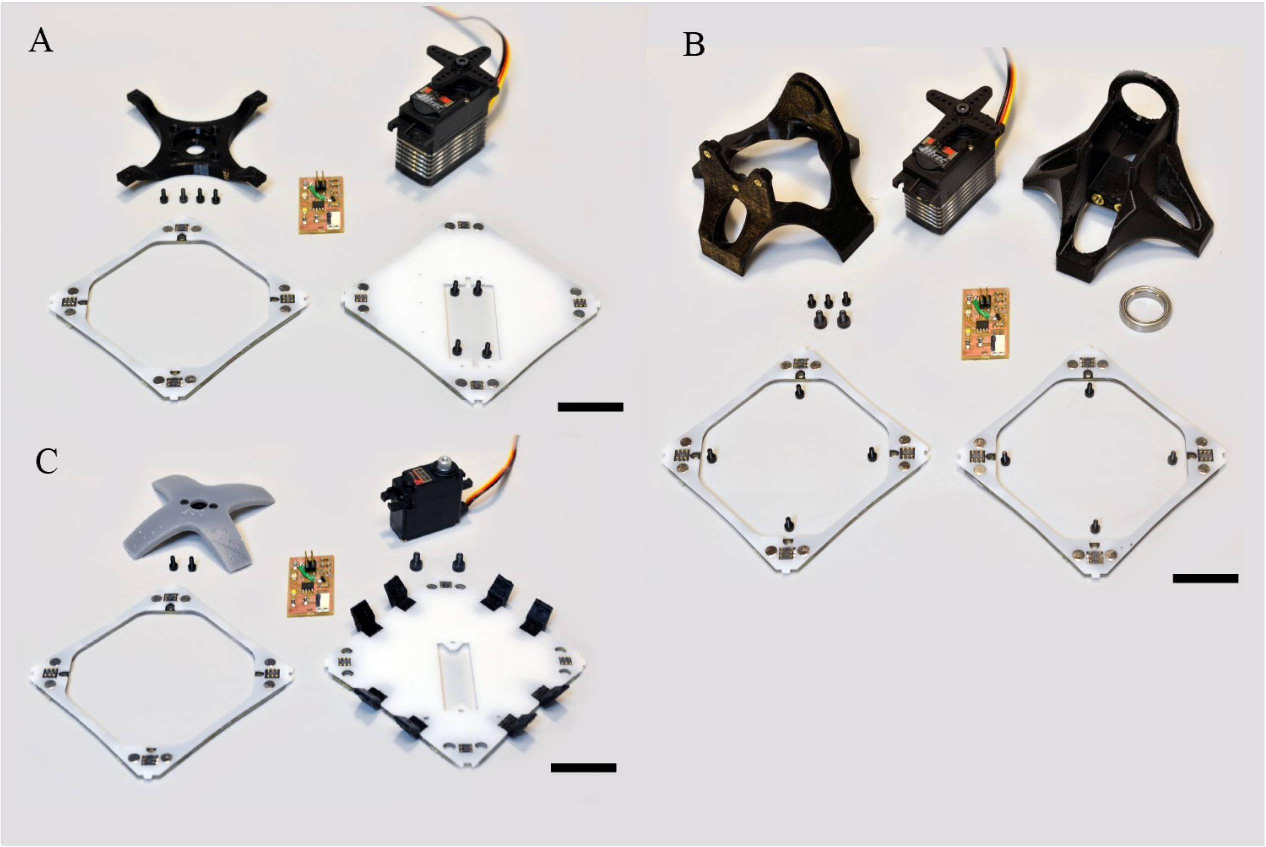

Figure S2. **Actuator Components.** **(A)** Wrist actuator, **(B)** Elbow Actuator, **(C)** Gripper (25 mm scale bars)

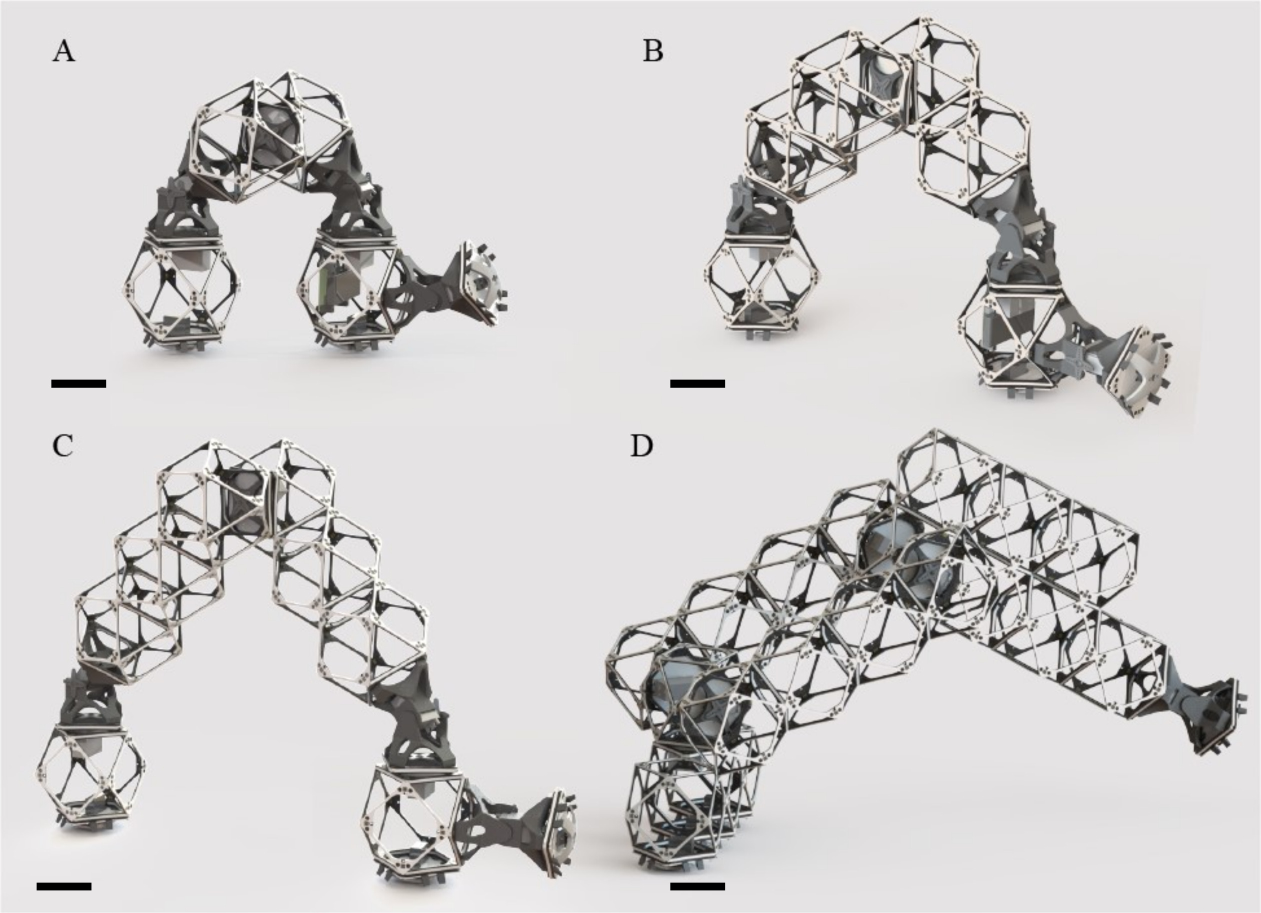

Figure S3. Hierarchical Robots Design Space. Example of carrier robots showing the design degrees of freedom (using the discrete robotic toolkit), either changing length or member stiffness. Example of carrier robots able to travel **(A)** one voxel per step, **(B)** 2 voxels per step, **(C)** 3 voxels per step, **(D)** or carry more passive or functional voxels (concept art showing stiffness can get added programmatically). (50 mm scale bars).

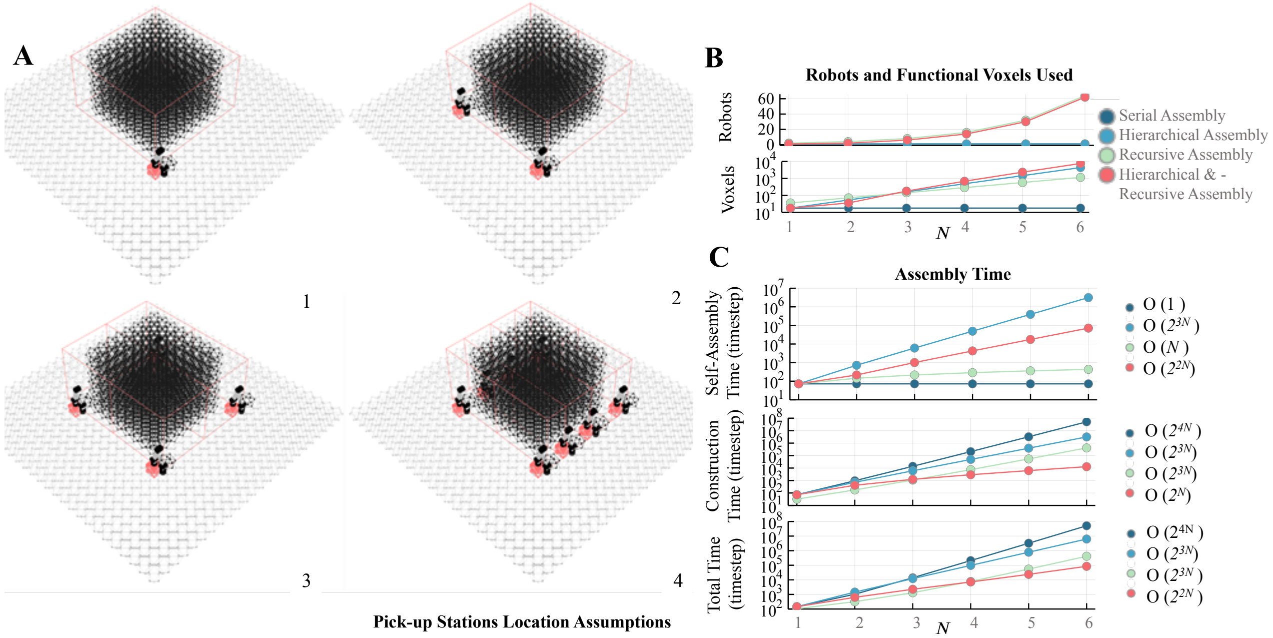

Figure S4. **Detailed Assumptions for Comparative Cube Construction Study.** **(A)** Assumptions for Pickup Location and Building Boundary **(1)** 1 Robot, **(2)** 2^1 Robots, **(3)** 2^2 Robots, **(D)** 2^3 Robots. **(B)** Number of Robots and Functional Voxels Used, **(C)** Self-Assembly, Construction and Total Time.

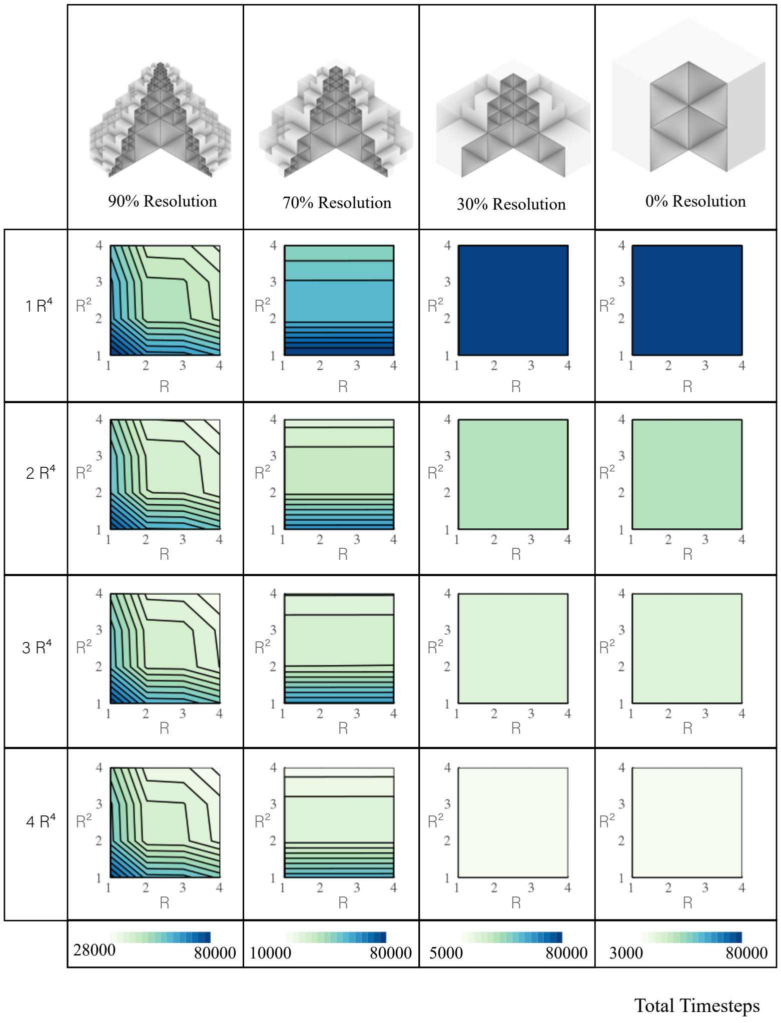

Figure S5. Effects of Structure Resolution on Total Construction Time. Total construction time needed to build a target shape (cone) with different resolutions using 1, 2, 3 and 4 robots of size R, R^2 and R^4.
