# Supplementary material for: Self-replicating hierarchical modular robotic swarms
Source: Commun Eng. 2022 Nov 22;1:35. doi: 10.1038/s44172-022-00034-3 (PMC10955888; doi:10.1038/s44172-022-00034-3)
Supplement: Supplementary file 3 — Description of Additional Supplementary Files [file 44172_2022_34_MOESM3_ESM.docx]

Description of Additional Supplementary Files

**File name:** Supplementary Movie 1

**Description:** Video showing the discrete robotic toolkit, carrier robot assembly, reconfigurability, the carrier robot walking on the lattice, and it removing and placing voxels..

**File name:** Supplementary Movie 2

**Description:** Using the developed simulation environment, we show how can a robot build another robot, and we demonstrate the capabilities of the discrete parametric design tool to design new hierarchical robot architectures.

**File name:** Supplementary Movie 3

**Description:** We first show how, given the same task of building a 4·4·4 robot, if the robot is able to build in a hierarchical manner the construction time will decrease by 40% as travel time decreases. Then the interactive interface for the adaptive shape compiler is shown, were all blocks that belong to the same bin can be built simultaneously. Finally, a simulation of path planning and assembly of a cone by a self-replicating hierarchical robotic swarm.
