# Supplementary material for: Self-replicating hierarchical modular robotic swarms
Source: Commun Eng. 2022 Nov 22;1:35. doi: 10.1038/s44172-022-00034-3 (PMC10955888; doi:10.1038/s44172-022-00034-3)
Supplement: Supplementary file 1 — Abdel-Rahman_PR File [file 44172_2022_34_MOESM1_ESM.pdf]

nature portfolio

Peer Review File

Self-Replicating Hierarchical Modular Robotic Swarms

Open Access This file is licensed under a Creative Commons Attribution 4.0 International License, which permits use, sharing, adaptation, distribution and reproduction in any medium or format, as long as you give appropriate credit to the original author(s) and the source, provide a link to the Creative Commons license, and indicate if changes were made. In the cases where the authors are anonymous, such as is the case for the reports of anonymous peer reviewers, author attribution should be to 'Anonymous Referee' followed by a clear attribution to the source work. The images or other third party material in this file are included in the article's Creative Commons license, unless indicated otherwise in a credit line to the material. If material is not included in the article's Creative Commons license and your intended use is not permitted by statutory regulation or exceeds the permitted use, you will need to obtain permission directly from the copyright holder. To view a copy of this license, visit http://creativecommons.org/licenses/by/4.0/.

Reviewers' comments:

Reviewer #1 (Remarks to the Author):

Review of "Recursive Robotic Swarms"

This paper examines a critical area of reconfigurable systems: how to quickly scale up a robotic workforce and use it to efficiently assemble materials into a desired structure.
This assumes that
(1) the system has a lattice substrate sufficiently large to build the desired structure
(2) there is a central intelligence that can communicate sets of instructions to individual robots
(3) the system starts with one small robot
(4) the system has a supply of robot components that are delivered to a set location of the workspace at the desired rate.
(5) there is a sufficient supply of voxel cubes that appear when needed at special depot locations of the workspace.
(6) the robots can assemble and disassemble the cubes by laying them in place, or by lifting them apart.

The paper demonstrates a hardware system that reasonably approximates these assumptions.
The bulk of the paper is dedicated to explaining a scalable robot design that can be built with one or more small robots, and a software scheme that compares total assembly times based on different compositions of robots. The paper ends with an optimization scheme that decided what robots to construct, and in what order to construct the desired shape.

My favorite part of the paper is that it attacks the big problem of optimal construction with reconfigurable robots, shows a non-trivial solution that builds at O(2^(2N)) more voxels per timestep, and relates the insights gained to biology.

It is wonderful how complete the paper is, from including weight and cost of all the robot materials, the careful construction and algorithms for the robots, to the beautiful 3 videos.

"BIG ISSUES" In order of importance

The strength of the robots as described grows linearly with the dimension of the robots (extra actuators are stacked as four-bar linkages). However, the weight of the robot and the weight of components to pick up grows cubicly with the dimension of the robot. this isn't that worrisome, because the robot and the components are made from ultra-light voxels. What is concerning is that the attachment force of the cubes grows with the square of the dimension. Separating voxels that are magnetically connected is hard. When a 4x4 cube must be picked up, all 16 cubes in contact with the substrate need to have their magnetic bounds broken. How can this be overcome? Perhaps the cubes can be separated by peeling from one edge, so only a number of bonds linear in the dimension of the robot need to be broken?

No other big issues. There are a number of minor issues that will improve the paper

"MINOR EDITS"

The title seems wrong. "Recursion" typically solves a big problem where the solution depends on solutions to smaller instances of the same problem. A more accurate title would be 'Hierarchal replicating robotic swarms". It doesn't have the same ring to it.

line 7: please define recursion. The supplied definition "ribosomes can make ribosomes" sounds like

self-replication, not recursion.

Line 16 -- should be "replication" not recursion. (One can argue that the Termes project was doing recursion -- the termes robots place one block, and then call the same solver on the slightly simpler problem).

50: should the gantry take max(x,y,z) time since each axis is independent? Or is the z time applied first to avoid collisions? Regardless, why does the time have a sqrt(x,y) term? Most gantries have independent axes for x and y.

50: In both line 51 and line 53, all the summations need to sum from 1 to 2^N instead of from 1 to N. As written, the timing for construction for the construction time gives (3 d)/(F S)*(N^3 + N^4), not (3 d)/(F S)*(2^(4*N) + 2^(3*N)). This is a large discrepancy that is off by a factor of (8^n + 16^n)/(n^3 + n^4).

50: It seems a bit strange that the factor F for slow down is in the denominator, which means that F needs to be a number smaller than 1. it would be more natural to have it in the numerator.

Fig. 1, (due to the writing above, I expected to see an entry for the gantry, but did not see it).
Fig. 1, B please arrange the legend entries to correspond with the top to bottom ordering of the plots, otherwise it is needlessly hard to reference.
Fig. 1 B, the legend does not have 2^{-N} in math mode, which makes the '-' sign look like a hyphen, rather than a math minus sign. Also neither N is in math mode.
Fig. 1 C, What is a 'resolvable' component? Without a definition, this is impossible to verify.

Many of the plots are not vector graphics, but certainly deserve to be. It is relatively easy to save a line plot as a vector graphic, which should be the norm for academic publishing. All the annotations (arrows and text) should also be vectors. As a corollary, for many of the images higher resolution would be nice (it is hard to make out details in the electrical connections in Fig. 2 C).

Fig. 3 B the figures shown seem wrong. The R robot has 1 voxel long arm segments, but the R2 and the R4 both have three voxel-long arm segments. Perhaps the R2 should have 2 voxel arms, and the R4 should have 4 voxel arms? This would allow them to take step sizes that scale with their footprint.

128--129 introduces new terminology. What is L2? L2.5? L3?

Fig 4, This figure is gorgeous. However, the subdivision in A doesn't match the division in B and C. It would be better for them to match.
Fig. 4C, what is the meaning of the dashed circles? What does it mean that blocks are in the same bin? Perhaps blocks in one bin could be circled as an example. Is SDF related to the dashed circles, the thin lines, or the color?

Fig 4 and 5 should be vector graphics. They are lovely, but suffer from low resolution.

Fig. 7 the robots shown do not match those in Fig. 5 or Fig. 7. It would make the paper easier to follow if the same robot types were used throughout.

"TINY EDITS"

Very often the variables are not in math mode. For instance, S in line 50, d in line 47, 62 'N' two times, 66 'H', 68, 69 'N'... 'S" in 149, T in 155, 157 (why not use \pm for +/-?). 'H' in Fig. 5 caption

53, missing space after ')'.

57, remove comma after "carrier robot,"

61 'fist' -> 'first'

66 remove comma after 'to carry,'
67 'block' -> 'blocks' (or should it be 'cubes'?)
76 'that we were" -> "were we"
78 'large scale' -> 'large-scale'

Fig. 2 caption 'pcb' -> 'PCB' (and many other locations, since PCB is an acronym, and is capitalized in a few places).

95 & 97 -- please give dimensions in metric. English measurements can be given in parenthesis afterwards. cm is not preferred, since it isn't a standard (https://standards.ieee.org/wp-content/uploads/import/governance/revcom/scc14.pdf). Also, the cubed notation should be a superscript.

148 "task specific" => "task-specific"

177 "underhangs" not under-hangs

197 R^4 should be (H=3), not H=2.
Fig. 6 caption has two periods at the end, should have just 1.
219 \cdot not '*'.
236 is not vs isn't
238 "off-the-shelf"

References: many of these are missing years, which would be helpful to understand the evolution of the field

"Evolve","Reproduce", "Expand" gives two 'E's. Maybe 'Construct' or 'Assemble' or 'build'?

356 please add line numbers for the algorithms.

Algorithm 2, please use math mode for variables.
Alg 2 "place b it at target location' -> "place it at target location'

table S1 please add units for weight (and state that prices are in USD) --- this was done for the gripper, so maybe the table was shuffled inadvertently.

table S1, the bottom total has too many decimal places.

357 PCB not pcb
FIg. S3, space before "(D)". These pictures are lovely, but don't overlap with the robots in Fig. 3. It would be helpful to have them represented here. Also, Fig S3 D is incomprehensible to me -- I can't resolve where the actuators are, nor how many there are. It would help to have a subcaption for each listing the number of each type of component.

Fig. S4 caption delete "Time Assembly" in last 3 words.

370 X,Y,Z in math mode.(many other variables after this)

VIDEO:

The video editing is lovely. Please add a scale bar for the size of the components
Video 1 -- the robot removes a voxel, but never places one. It would be nice to run the video 2 seconds longer and show the voxel being placed.
Video 2: 0:45 "Discrete" not "Dicrete
Video 3: 0:04 "Hierachal" -- "Hierarchical"
Video 3 is quite pleasing. At 1:08 it appears that the large robot is intersecting the 4x4 cube. Many other interactions might also be in collision, but that is hard to tell at this video speed. This doesn't disprove any point in the paper since the simulation is an abstraction, but if it is fixable that would be nice.

-Aaron Becker

Reviewer #2 (Remarks to the Author):

The authors propose a reconfigurable robotic system composed of building blocks, with construction applications as main target, to realize structures serially, recursively, and hierarchically. The Authors build on their previous work (ref. 25 in the manuscript).

In summary, I found the description not so convincing. Based on the current text it is hard for me to assess the significance of the proposed contribution with respect to ref. 25. I had the feeling that the advancements were minor and that the motivation behind the new paper is not clear enough. I strongly believe that aspects of structural integrity, and mechanical subsystem actuation/motion feasibility (both static and dynamic with actuation and power limits) -now left by the authors for future work as mentioned on page 6- would be an important part to consider for making the work stronger and more convincing for real applicability.

Other comments:

- In the abstract it is mentioned that high performance cellular structures are a motivating application. It was not clear to me what in the performance metric the authors are considering.

- In the abstract "joint discretization" is mentioned but I find this sentence unclear.

- I think that the introduction does not provide a clear motivation for the proposed work. Also, it does not provide a clear description of advancements with respect to ref. 25.

- Lines 35-37. I find this sentence debatable. I would argue that robots are by nature multi-function machines.

- I would suggest the authors to describe more formally the addressed problems in the introduction and directly motivate the significance for their solution.

- On line 59 assumptions are mentioned referring to a Figure. However, I could not find any assumption described/formulated in the text. I think assumptions should be more clearly formulated/described in the main text along with their justification.

- Units should always be indicated (doublecheck Table S1). Also, doublecheck consistency for the metric system (see e.g., inch on line 95)

- On lines 150-152. Why did the authors need to consider a spatial-temporal Markov model to predict

and store the state of the system? What is the motivation behind this choice? Were there alternatives?

- I would expect the results to be presented in a more generic way with more detailed algorithms descriptions and systematic procedures that a reader interested in replicating or building on these results could follow.

Comments on Video -Movie S1 and S3:
I think that a description with more insights of what is shown in the video would improve it.

Reviewer #3 (Remarks to the Author):

The authors introduce a material-robot system that can be used to create robotic swarms either in a serial, recursive or hierarchical way.
The system makes use of active lattice voxels as primary building block of the robots, and passive voxels as structural components. The composed robots can in turn be used to build voxel-based structures. By exploiting recursion and hierarchy they are able to reach an assembly thorughput comparable to that of ribosomes.

The results are well presented and the implementation details included both in the text and in the supplementary material should allow the reader to succesfully reproduce the work.
The authors tackle a complex problem by presenting a novel algorithm that optimizes at the same time the building sequence of the desired shape, the swarm configuration and the task allocation problem.
While the algorithm might be a bit "custom" to the application, the results are quite impressive.
Future works, as correctly stated also by the authors, could take into account also the robot morphology and the pickup locations as variables to be optimized, which could make the algorithm much more general and open up many scenarios also in different fields.

Some minor comments on things that could be a bit more clear:

- The title could be a bit less general and a bit more indicative of the scope of the paper. The algorithm presented here focuses on a discrete material-robot system used for construction, which would not translate right away to robotic swarms used for other applications (as the title might hint). It could be good to give the reader a better idea of what to expect from the paper.

- It could make sense to include some more definitions in the introduction. Concept like 'recursion', 'hierarchy', 'material-robot systems', 'carrier and assembler robot', etc. are all terms that could be better introduced at the beginning of the paper, to facilitate reading.

- The number of robots to use in a recursive swarm strategy seems a very important variable to optimize when trying to maximize the assembly throughput. In fact this number is selected by the algorithm as in the 'swarm configuration' section and fig.5. Why on page 2 when considering the throughput of recursive swarms the number is fixed to 2^N? Wouldn't the beahviour (speed) change for an higher number of twins? It would be good to provide some more reasoning behind this assumption.

COMMSENG-22-0033: Response to Referees

Reviewers' comments:

We thank the reviewers very much for their time and detailed feedback. We appreciate the opportunity to discuss the paper and use their suggestions to ultimately improve it.

Reviewer #1 (Remarks to the Author):

We wanted again to thank reviewer 1. We are very grateful for this very detailed, thoughtful, and helpful review.

Review of "Recursive Robotic Swarms"

This paper examines a critical area of reconfigurable systems: how to quickly scale up a robotic workforce and use it to efficiently assemble materials into a desired structure.
This assumes that
(1) the system has a lattice substrate sufficiently large to build the desired structure
(2) there is a central intelligence that can communicate sets of instructions to individual robots
(3) the system starts with one small robot
(4) the system has a supply of robot components that are delivered to a set location of the workspace at the desired rate.
(5) there is a sufficient supply of voxel cubes that appear when needed at special depot locations of the workspace.
(6) the robots can assemble and disassemble the cubes by laying them in place, or by lifting them apart.

The paper demonstrates a hardware system that reasonably approximates these assumptions.
The bulk of the paper is dedicated to explaining a scalable robot design that can be built with one or more small robots, and a software scheme that compares total assembly times based on different compositions of robots. The paper ends with an optimization scheme that decided what robots to construct, and in what order to construct the desired shape.

This is a very concise summary of the paper and our contribution.

My favorite part of the paper is that it attacks the big problem of optimal construction with reconfigurable robots, shows a non-trivial solution that builds at O(2^(2N)) more voxels per timestep, and relates the insights gained to biology.

We really appreciate the reviewer’s comment. This is indeed one of the main objectives of the paper.

It is wonderful how complete the paper is, from including weight and cost of all the robot materials, the careful construction and algorithms for the robots, to the beautiful 3 videos.

We thank the reviewer very much for the supportive remarks.

"BIG ISSUES" In order of importance

The strength of the robots as described grows linearly with the dimension of the robots (extra actuators are stacked as four-bar linkages). However, the weight of the robot and the weight of components to pick up grows cubicly with the dimension of the robot. this isn't that worrisome, because the robot and the components are made from ultra-light voxels. What is concerning is that the attachment force of the cubes grows with the square of the dimension. Separating voxels that are magnetically connected is hard. When a 4x4 cube must be picked up, all 16 cubes in contact with the substrate need to have their magnetic bounds broken. How can this be overcome? Perhaps the cubes can be separated by peeling from one edge, so only a number of bonds linear in the dimension of the robot need to be broken?

That's an interesting question. We first wanted to reiterate on the point raised in the discussion (line 213-217) that even though the system shown uses magnetic connections between the voxels, joints with improved stiffness and strength will be required for continuum higher-performance structures as well for scaling up the hierarchical robots. The plan is to use mechanical joints, bistable latches, and for weaker magnets to be only used for alignment purposes. Pinned joints also can be used similar to Ref. 15 to separate the joining force from the orthogonal degrees of freedom, and complexity could be added to the robots to assemble these joints .

Concerning the point raised about the detachment force, since the algorithm presented is only concerned with construction and not reconfiguration, the only two instances where the blocks need to be detached are when they are picked up from the pickup stations or when they are detached from the robot itself (the gripper).
In both cases, the magnets of the substrate around the pickup station and the robot gripper could be designed to be much weaker than ones in the voxels building material (the rotating latch in the robot's gripper is the mechanism that's holding the blocks and not the magnet). Hence, detaching the blocks should not be a concern for the construction algorithm. Alternative smart joint mechanisms, as stated before, should be used if one needs to generalize to structure reconfiguration instead of just construction.

No other big issues. There are a number of minor issues that will improve the paper

"MINOR EDITS"

The title seems wrong. "Recursion" typically solves a big problem where the solution depends on solutions to smaller instances of the same problem. A more accurate title would be 'Hierarchal replicating robotic swarms". It doesn't have the same ring to it.

This is a good point. We agree with the reviewer and we changed the title to "Self-Replicating Hierarchical Robotic Swarms".

line 7: please define recursion. The supplied definition "ribosomes can make ribosomes" sounds like self-replication, not recursion.

We agree with the reviewer that self-replication is a better choice of wording than recursion (hence it was replaced in all instances in the article). The word recursive however was left in the document as we are using it according to its mathematical definition: "relating to or involving the repeated application of a rule, definition, or procedure to successive results" (oxford dictionary).

Line 16 -- should be "replication" not recursion. (One can argue that the Termes project was doing

recursion -- the termes robots place one block, and then call the same solver on the slightly simpler problem).

As stated in the previous comment, we agree with the reviewer and we replaced in all instances of the term recursion with self-replication.

50: should the gantry take max(x,y,z) time since each axis is independent? Or is the z time applied first to avoid collisions? Regardless, why does the time have a sqrt(x,y) term? Most gantries have independent axes for x and y.

The reviewer is correct, the z time applied first to avoid collisions, hence we changed the equation to $max(x,y)+z$. The reason we had the $sqrt(x,y)$ term is that we were following reference 23 equation 1 where the speed to go to a point x,y,z was set as the shortest path $sqrt(x^2+y^2+z^2)$, which assumes the gantry will go from point A to B in a straight line with constant speed.

50: In both line 51 and line 53, all the summations need to sum from 1 to 2^N instead of from 1 to N. As written, the timing for construction for the construction time gives (3 d)/(F S)*(N^3 + N^4), not (3 d)/(F S)*(2^(4*N) + 2^(3*N)). This is a large discrepancy that is off by a factor of (8^n + 16^n)/(n^3 + n^4).

Thank you for catching this typo, we corrected both equations.

50: It seems a bit strange that the factor F for slow down is in the denominator, which means that F needs to be a number smaller than 1. it would be more natural to have it in the numerator.

We agree with the reviewer and changed F to be a number smaller than 1 (line 54) and edited the corresponding equations.

Fig. 1, (due to the writing above, I expected to see an entry for the gantry, but did not see it).

We added an entry for the gantry (that is 10x speed) as requested. The reason it was not added is that it will more or less have the same trend as the serial construction curve, and will just have an offset corresponding to the speedup factor. We added it though for completeness.

Fig. 1, B please arrange the legend entries to correspond with the top to bottom ordering of the plots, otherwise it is needlessly hard to reference.
Fig. 1 B, the legend does not have 2^{-N} in math mode, which makes the '-' sign look like a hyphen, rather than a math minus sign. Also neither N is in math mode.
Fig. 1 C, What is a 'resolvable' component? Without a definition, this is impossible to verify.

Thank you for your comments, in Fig. 1 we arranged the legend entries, changed 2^{-N} and N to be in math mode and replaced “resolvable element” to be “amino acid part”.

Many of the plots are not vector graphics, but certainly deserve to be. It is relatively easy to save a line plot as a vector graphic, which should be the norm for academic publishing. All the annotations (arrows and text) should also be vectors. As a corollary, for many of the images higher resolution would be nice (it is hard to make out details in the electrical connections in Fig. 2 C).

We thank the reviewer for the suggestion, we have indeed replaced all figures (in the main text and supplementary section) with high resolution vector graphics.

Fig. 3 B the figures shown seem wrong. The R robot has 1 voxel long arm segments, but the R2 and the R4 both have three voxel-long arm segments. Perhaps the R2 should have 2 voxel arms, and the R4 should have 4 voxel arms? This would allow them to take step sizes that scale with their footprint.

We have addressed this comment and changed the design of the R2 and R4 robots in figure 3B and the rest of the document (calculations and simulation results).

128--129 introduces new terminology. What is L2? L2.5? L3?

Thank you for pointing this out. We have added in the figure caption an explanation for L0 to L14 (layer number) and S1, S2, S4 (block size).

Fig 4, This figure is gorgeous. However, the subdivision in A doesn't match the division in B and C. It would be better for them to match.

We thank the reviewer for the supportive remark, we have changed in Fig. 4 A the last two subdivisions to march B and C. That indeed helps the reader understand the process better.

Fig. 4C, what is the meaning of the dashed circles? What does it mean that blocks are in the same bin? Perhaps blocks in one bin could be circled as an example. Is SDF related to the dashed circles, the thin lines, or the color?

We added a legend in Fig. 4C to explain the dashed circles. They explain the different branching, where the blocks are sorted first based on the layer number, block size and finally SDF value (hence bin).

Fig 4 and 5 should be vector graphics. They are lovely, but suffer from low resolution.

The reviewer is correct, we have replaced them to be higher resolution vector graphics.

Fig. 7 the robots shown do not match those in Fig. 5 or Fig. 7. It would make the paper easier to follow if the same robot types were used throughout.

The robots in Fig. 7 does match the ones in Fig 3 and 5. We thought the misunderstanding might be because we only showed a front view of the robots in Fig. 3 B. Hence we added a side perspective view for the readers to understand the “depth” of the robots.

"TINY EDITS"

Very often the variables are not in math mode. For instance, S in line 50, d in line 47, 62 'N' two times, 66 'H', 68, 69 'N'... 'S" in 149, T in 155, 157 (why not use \pm for +/-?). 'H' in Fig. 5 caption

Thanks a lot for pointing out these types. We have corrected them.

53, missing space after ')'.

We have corrected this.

57, remove comma after "carrier robot,"

Corrected.
61 'fist' -> 'first'
Corrected.
66 remove comma after 'to carry,'

Corrected.
67 'block' -> 'blocks' (or should it be 'cubes'?)

Corrected.
76 'that we were" -> "were we"

Corrected.
78 'large scale' -> 'large-scale'
Corrected.
Fig. 2 caption 'pcb' -> 'PCB' (and many other locations, since PCB is an acronym, and is capitalized in a few places).
Corrected.
95 & 97 -- please give dimensions in metric. English measurements can be given in parenthesis afterwards. cm is not preferred, since it isn't a standard (https://standards.ieee.org/wp-content/uploads/import/governance/revcom/scc14.pdf). Also, the cubed notation should be a superscript.

Thanks for pointing this out. We have corrected it.

148 "task specific" => "task-specific"

Corrected.

177 "underhangs" not under-hangs
Corrected.
197 R^4 should be (H=3), not H=2.

Corrected.
Fig. 6 caption has two periods at the end, should have just 1.

Corrected.
219 \cdot not ''.*

Corrected.
236 is not vs isn't

Corrected.
238 "off-the-shelf"

Corrected.

References: many of these are missing years, which would be helpful to understand the evolution of the field

Thanks for the comment. We have edited them to include the years.

"Evolve","Reproduce", "Expand" gives two 'E's. Maybe 'Construct' or 'Assemble' or 'build'?

That makes sense. We replaced "Expand" with "Build".

356 please add line numbers for the algorithms.

Done.

Algorithm 2, please use math mode for variables.

Done.

Alg 2 "place b it at target location' -> "place it at target location'
This has been corrected as well.

table S1 please add units for weight (and state that prices are in USD) --- this was done for the gripper, so maybe the table was shuffled inadvertently.
This has been changed accordingly.
table S1, the bottom total has too many decimal places.
Corrected.
357 PCB not pcb

Corrected.
FIg. S3, space before "(D)". These pictures are lovely, but don't overlap with the robots in Fig. 3. It would be helpful to have them represented here. Also, Fig S3 D is incomprehensible to me -- I can't resolve where the actuators are, nor how many there are. It would help to have a subcaption for each listing the number of each type of component.

Good point. However, the purpose of the figure is not to overlap with Fig. 3 but just concept art to show the design degrees of freedom (using the discrete robotic toolkit), by either varying the length or member stiffness to carry more passive or functional voxels (Fig S3 D). We adjusted the caption to further explain this point.

Fig. S4 caption delete "Time Assembly" in last 3 words.
Corrected.
370 X,Y,Z in math mode.(many other variables after this)
Corrected.

VIDEO:

The video editing is lovely. Please add a scale bar for the size of the components

We thank the reviewer for his comments. A scale bar has been added in the bottom right of the first video.
Video 1 -- the robot removes a voxel, but never places one. It would be nice to run the video 2 seconds longer and show the voxel being placed.

Thank you for pointing this out. We edited the video accordingly.
Video 2: 0:45 "Discrete" not "Dicrete

Corrected.
Video 3: 0:04 "Hierachal" -- "Hierarchical"

Corrected.
Video 3 is quite pleasing. At 1:08 it appears that the large robot is intersecting the 4x4 cube. Many other interactions might also be in collision, but that is hard to tell at this video speed. This doesn't disprove any point in the paper since the simulation is an abstraction, but if it is fixable that would be nice.

Thanks a lot for pointing this out. We have indeed re-ran the simulation and changed the starting point/pickup station for the largest robot to avoid the collision.

-Aaron Becker

Reviewer #2 (Remarks to the Author):

The authors propose a reconfigurable robotic system composed of building blocks, with construction applications as main target, to realize structures serially, recursively, and hierarchically. The Authors build on their previous work (ref. 25 in the manuscript).

This is an accurate summary of the work.

In summary, I found the description not so convincing. Based on the current text it is hard for me to assess the significance of the proposed contribution with respect to ref. 25. I had the feeling that the advancements were minor

We thank the reviewer for the comment, however the focus of this paper is very different than ref. 25. In ref. 25, we introduced a monolithic carrier robot that is able to walk on the structure and error correct to build discrete cellular structures. In contrast, in this paper, as the reviewer stated, we introduce a “reconfigurable robotic system” that is made from the structure itself (hence the robot and the structure it is building are indistinguishable). With the new robotic system, the robots are able to not only build structures but build other robots of different sizes (which definitely wasn’t possible in ref. 25). Even though we built upon the algorithm introduced in ref. 25 for serial and swarm assembly, we introduced an new algorithm, a new path planning and task allocation strategy for recursive and hierarchical assembly, as well as novel scalable shape compiler for scalable hierarchical assembly.

and that the motivation behind the new paper is not clear enough.

The main motivation of the paper, as started in the first two paragraphs of the introduction, is to address the scaling of construction automation and collective robotic construction, which scale poorly (either in

precision, cost or speed) with the size of the construction. Inspired by principles from biological growth (self-replication and hierarchy), we introduce a reconfigurable robotic system that can precisely build large structures, with a throughput that increases with the size of the system that is desired to be built. To the best of our knowledge this is a new contribution to the literature as there is no prior art about this subject matter.

I strongly believe that aspects of structural integrity, and mechanical subsystem actuation/motion feasibility (both static and dynamic with actuation and power limits) -now left by the authors for future work as mentioned on page 6- would be an important part to consider for making the work stronger and more convincing for real applicability.

We agree with the reviewer about the importance of further developing the structural integrity, and mechanical subsystem actuation of the system. However, concerning the first point, adjusting the path planning algorithm to take into consideration the structural integrity of the system built (page 6 line 163), will not have any effect on the calculations and analysis done in Figure 1, 4, 5 & 6, since we are building the structures layer by layer from bottom to top. It will only matter when building spanning structures, and that will also not alter the relative construction time and the speedup that recursive and hierarchical assembly offer.

Concerning the mechanical subsystem actuation/motion feasibility, we believe, as reviewer 1 stated, that we presented "a hardware system that reasonably approximates [our] assumptions". The main point of the paper was to explain the importance of self-replication and hierarchy to build scalable collective robot construction systems. The hardware presented is an instantiation of such a system, and used as a starting point to do the scaling calculations/analysis presented. These calculations/simulations though would not change once these aspects are addressed in future work, on the contrary the work presented serves as a guideline for the requirements needed for a new hardware generation.

Other comments:

- In the abstract it is mentioned that high performance cellular structures are a motivating application. It was not clear to me what in the performance metric the authors are considering.

We have indeed edited the abstract and specified that we mean high specific modulus and strength. This is also further explained in the introduction (line 27-28) and the "material-robot system" section (line 92).

- In the abstract "joint discretization" is mentioned but I find this sentence unclear.

We edited the abstract and changed it to just discretization. We are referring to the material discretization mentioned in line 1.

- I think that the introduction does not provide a clear motivation for the proposed work. Also, it does not provide a clear description of advancements with respect to ref. 25.

We touched upon this point in our previous response. We hope that we further explained our motivation and the difference between the work presented here and ref. 25. We are happy though to further address this point if the reviewer has more specific concerns.

- Lines 35-37. I find this sentence debatable. I would argue that robots are by nature multi-function machines.

Good point. We didn't mean that all robots have a single function, we meant fixed-functions (hence not

flexible). However, we understand the confusion hence we changed “fixed-function robots” to “monolithic robots”.

- *I would suggest the authors to describe more formally the addressed problems in the introduction and directly motivate the significance for their solution.*

We explain the main problems that we are addressing in the first paragraph. We added a sentence in line 10-11 to explain the motivation of the paper and directly motivate the significance for their solution.

- *On line 59 assumptions are mentioned referring to a Figure. However, I could not find any assumption described/formulated in the text. I think assumptions should be more clearly formulated/described in the main text along with their justification.*

We have a detailed explanation of the assumptions in supplementary section S4 (we adjusted the reference to refer to the section and not to the figure). We really believe that the assumptions, if added to the main text will distract the readers on the main objective of the papers and the results.

- *Units should always be indicated (doublecheck Table S1). Also, doublecheck consistency for the metric system (see e.g., inch on line 95)*
We thank the reviewer for pointing this out. We have indeed addressed that in the edits.

- *On lines 150-152. Why did the authors need to consider a spatial-temporal Markov model to predict and store the state of the system? What is the motivation behind this choice? Were there alternatives?*

The spatial-temporal of the model was used for path planning and collision avoidance. A Markov model was used as the future state of the system only depends on the current state and not the past states. That was chosen to make the model simple and flexible in case there are changes in the system (line 161 – changes in the number of robots or pickup stations). The stochasticity of the Markov model is controlled by safety factor and desired fault tolerance (line 158). If no safety factor to fault tolerance is considered then we use a deterministic model.

We however discussed in line 228 that we are not claiming that the algorithm presented is optimal; more complex path planning and collision avoidance strategies could be implemented to decrease the construction time (a more detailed study and algorithm comparison is in ref. 39).

- *I would expect the results to be presented in a more generic way with more detailed algorithms descriptions and systematic procedures that a reader interested in replicating or building on these results could follow.*
We appreciate the reviewer’s comment, we think we explained the algorithms, the assumptions needed to reproduce the results. Both reviewer 1 and 3 stated that the paper is “complete” and that “the results are well presented and the implementation details included both in the text and in the supplementary material should allow the reader to successfully reproduce the work”. We are willing to edit/add any details if there are specific comments, or if you point out which part of the algorithm/procedure is unclear.

Comments on Video -Movie S1 and S3:
I think that a description with more insights of what is shown in the video would improve it.
Good Point. We added a detailed description of the video contents in line 356-364.

Reviewer #3 (Remarks to the Author):

The authors introduce a material-robot system that can be used to create robotic swarms either in a serial, recursive or hierarchical way.
The system makes use of active lattice voxels as primary building block of the robots, and passive voxels as structural components. The composed robots can in turn be used to build voxel-based structures. By exploiting recursion and hierarchy they are able to reach an assembly thorughput comparable to that of ribosomes.

This is indeed an accurate summary of the research.

The results are well presented and the implementation details included both in the text and in the supplementary material should allow the reader to succesfully reproduce the work.
The authors tackle a complex problem by presenting a novel algorithm that optimizes at the same time the building sequence of the desired shape, the swarm configuration and the task allocation problem.
While the algorithm might be a bit "custom" to the application, the results are quite impressive.
Future works, as correctly stated also by the authors, could take into account also the robot morphology and the pickup locations as variables to be optimized, which could make the algorithm much more general and open up many scenarios also in different fields.

We thank the reviewer for their supportive remarks and insightful comments.

Some minor comments on things that could be a bit more clear:

- The title could be a bit less general and a bit more indicative of the scope of the paper. The algorithm presented here focuses on a discrete material-robot system used for construction, which would not translate right away to robotic swarms used for other applications (as the title might hint). It could be good to give the reader a better idea of what to expect from the paper.

This is a good point, as reviewer 1 also suggested, we changed the title to “Self-Replicating Hierarchical Robotic Swarms”.

- It could make sense to include some more definitions in the introduction. Concept like 'recursion', 'hierarchy', 'material-robot systems', 'carrier and assembler robot', etc. are all terms that could be better introduced at the beginning of the paper, to facilitate reading.

Thank you for the suggestion, we have indeed added definitions for these terms in the introduction (line 24, 34, 40).

- The number of robots to use in a recursive swarm strategy seems a very important variable to optimize when trying to maximize the assembly throughput. In fact this number is selected by the algorithm as in the 'swarm configuration' section and fig.5. Why on page 2 when considering the throughput of recursive swarms the number is fixed to 2^N? Wouldn't the behavior (speed) change for an higher number of twins? It would be good to provide some more reasoning behind this assumption.

This is a very good question. When choosing the number of robots to build a cube of size 2^N, we were considering the underlying assumptions for the pickup locations explained in detail in Figure S4 and Section S4. As the reviewer said, the speed would increase when having a higher number of robots. However, the maximum number of robots that can be added to build a cube of size 2^N is 2*2^N, as there will be one robot/pickup station in front of each voxel at two opposing sides at the cube. That means that each robot is responsible for building a slice of width 1, length 2^N/2 and height 2^N.

The graphs below show both results (for R=2^N and R=2*2^N robots). Even though for the 2*2^N robots the throughput is higher for the smaller cubes, the trend will be the same where the complexity is O(1) we and the slope will decrease to 0 as the cube size increases.

Number of functional parts and assembly throughput for recursive assembly with R=2^N robots:

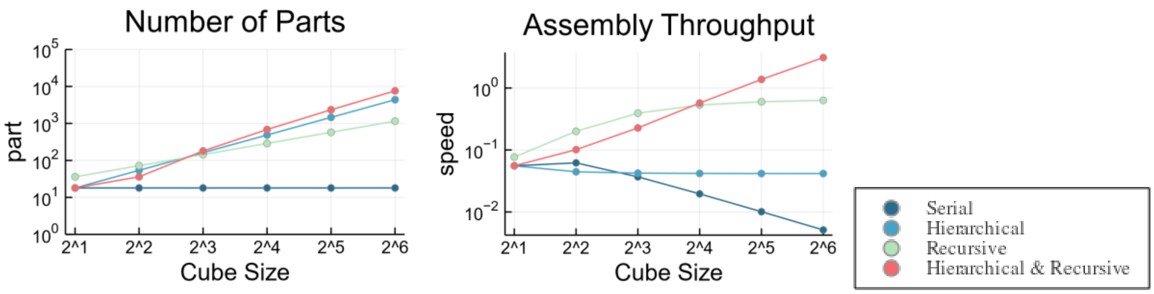

Number of functional parts and assembly throughput for recursive assembly (green) with R=2*2^N robots:

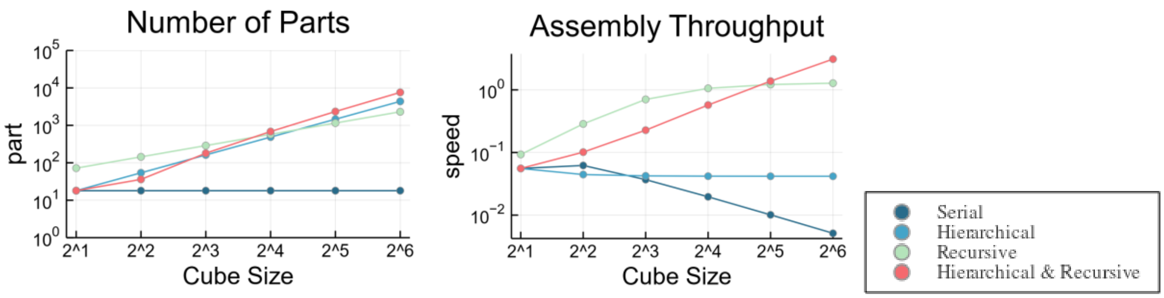

The reason we preferred to display the data for 2^N instead of 2*2^N was that, logistically, it is preferred to have a buffer (1 voxel) between each pickup station (each robot will be then responsible to build a slice of width 2, length 2^N/2 and height 2^N). That is especially useful to simplify the calculations and the path planning needed to prevent collision while the robots are turning back (as they need the neighboring voxel to turn). When there is a buffer they can all do U-turns simultaneously which simplifies the path planning.

We added a note about that point in line 372-373.

Reviewers' comments:

Reviewer #1 (Remarks to the Author):

Review of "Self-Replicating Hierarchical Robotic Swarms"

[my previous summary is still true]
"This paper examines a critical area of reconfigurable systems: how to quickly scale up a robotic workforce and use it to efficiently assemble materials into a desired structure.
This assumes that
(1) the system has a lattice substrate sufficiently large to build the desired structure
(2) there is a central intelligence that can communicate sets of instructions to individual robots
(3) the system starts with one small robot
(4) the system has a supply of robot components that are delivered to a set location of the workspace at
the desired rate.
(5) there is a sufficient supply of voxel cubes that appear when needed at special depot locations of the
workspace.
(6) the robots can assemble and disassemble the cubes by laying them in place, or by lifting them apart.
The paper demonstrates a hardware system that reasonably approximates these assumptions. The bulk of the paper is dedicated to explaining a scalable robot design that can be built with one or more small robots, and a software scheme that compares total assembly times based on different compositions of robots. The paper ends with an optimization scheme that decided what robots to construct, and in what order to construct the desired shape.

My favorite part of the paper is that it attacks the big problem of optimal construction with reconfigurable robots, shows a non-trivial solution that builds at O(2^(2N)) more voxels per timestep, and relates the insights gained to biology. It is wonderful how complete the paper is, from including weight and cost of all the robot materials, the careful construction and algorithms for the robots, to the beautiful 3 videos. "

The paper has been edited and improved. It is both timely and well executed, so it should be appreciated by the robotics community.

"BIG ISSUES" In order of importance

no big issues. The paper was revised.

"MINOR EDITS"

The text in Build Sequence Tree in Fig. 4 is still a bitmap, not a vector
Fig. S4, B and C the plots (but not the legends) are still bitmaps, not vector graphics

"Concerning the point raised about the detachment force, since the algorithm presented is only concerned with construction and not reconfiguration, the only two instances where the blocks need to be detached are when they are picked up from the pickup stations or when they are detached from the robot itself (the gripper)." --> but consider the large robot in Fig. 3, which is shown picking up a 4x4x4 voxel cube. This cube was presumably constructed by the smaller robots, so it would need to be picked up.

Line 13: "Teams of wheeled robot arms can collectively 3D print a single object larger than their individual workspace." The workspace for a single wheeled robot is unbounded. Perhaps this should be

"Teams of wheeled robot arms can collectively 3D print a single object larger than the workspace for any individual arm." Is there something that a team can do that a individual cannot in this example?

Line 131, please define L2.5. In figure 4, L means 'layer', but in 131-133 it seems to refer to an exponent term. Please also define L2 and L3.

In Fig. 6, what does X% resolution mean? how is 0% a cube, and 90% a more complicated step pyramid? Presumably there is a simple equation being used here.

"TINY EDITS" (e.g. typos)

Abstract: it seems awkward to describe swarm + object construction as "compile a shape". Is there a better way, e.g. "compose building components into both a swarm and the desired object"?
24: ", where the robots and the material they build are designed simultaneously and hence indistinguishable." -> ", where the robots and the material they build with are designed simultaneously and hence are indistinguishable." -- but is 'designed' the correct word here? Should this be "are designed to be indistinguishable.", since the purpose was to make the robot parts and building parts out of the same components?

Line 51, 52, x,by, z should be in math font
Line 57, N should be in math font

Fig. 1, A 'N' and 'H' should be in math font

Lines 63-66 are one long sentence. Please split this paragraph into several sentences.

Sentence 67 is awkward. 'but larger robots' -> but can also build larger robots'

Line 68 is also awkward. Perhaps remove word 'although' and clean this up.

Line 70, should 'N' be 'H'? Alternately, should lines 68-69 use 'N' instead?
Line 71, 'N' in math font

Line 77, 'E' in math font

Line 78, swap order of 'we were' to 'were we'

Lines 97 and 100, 102 please add spaces between number and units.

Caption Fig. 3, 'H' in math mode

Line 143 "A task entails picking up a building block from the pickup station in form of a list of high-level steps instructions towards the drop-off location" -> "A task entails picking up a building block from the pickup station in the form of a list of high-level step instructions towards the drop-off location"

222 please use \cdot rather than '.' for Nm unit.

51, 362, 382, 383, 384, 386, 388, 389 (and in eq S2) please use \cdot rather than '*' for multiplication

VIDEO:
these are great!

Reviewer #2 (Remarks to the Author):

The authors provided clarifications for all concerns I raised previously. However, I could not always find a place in the manuscript for some of the mentioned clarifications.

1) I found in the rebuttal the sentence
"Even though [...] we introduced an new algorithm, a new path planning and task allocation strategy for recursive and hierarchical assembly, as well as novel scalable shape compiler for scalable hierarchical assembly.",
but I could not find such a clear statement of novelty with respect to the previous work in the manuscript. I would have expected to find such a statement in the last paragraph of the introduction for example.

2) About the "aspects of structural integrity, and mechanical subsystem actuation/motion feasibility", I could not find the lines in the manuscript with the respective clarifications.

3) The sentence "I would suggest the authors to describe more formally the addressed problems in the introduction and directly motivate the significance for their solution." was not referring to a clarification to the top-level goal of the study, but to the technical problems faced instead -those that led to the novelties the authors mention in "1)" above-.

4) I could not find a "section 4" in the supplementary materials for the assumptions. I can find a "Figure S4" mentioning some assumptions followed by text (is this section 4?). Anyhow, the fact that the Authors assume not to have actuation and power limits in the proposed algorithms should be mentioned in the paper.

5) I would find it to be clearer if the algorithms that constitute the main novelty of the paper were presented with pseudocode and a description of said pseudocode. Anyhow, I also respect other reviewers' opinion as well as Author’s preference. Thus, feel free to keep the current presentation of the algorithms if that's your preference. However, it would be laudable if the Authors could share the code and data related to the main results as supplementary material or provide a link to a repository.

Reviewer #3 (Remarks to the Author):

The authors correctly answered all questions and doubts from the reviewer

COMMSENG-22-0033A: Response to Reviews

Reviewers' comments:

We thank the reviewers very much for their time and detailed feedback.

Reviewer #1 (Remarks to the Author):

Review of "Self-Replicating Hierarchical Robotic Swarms"

[my previous summary is still true]

"This paper examines a critical area of reconfigurable systems: how to quickly scale up a robotic workforce and use it to efficiently assemble materials into a desired structure.

This assumes that

(1) the system has a lattice substrate sufficiently large to build the desired structure

(2) there is a central intelligence that can communicate sets of instructions to individual robots

(3) the system starts with one small robot

(4) the system has a supply of robot components that are delivered to a set location of the workspace at the desired rate.

(5) there is a sufficient supply of voxel cubes that appear when needed at special depot locations of the workspace.

(6) the robots can assemble and disassemble the cubes by laying them in place, or by lifting them apart.

The paper demonstrates a hardware system that reasonably approximates these assumptions. The bulk of the paper is dedicated to explaining a scalable robot design that can be built with one or more small robots, and a software scheme that compares total assembly times based on different compositions of robots. The paper ends with an optimization scheme that decided what robots to construct, and in what order to construct the desired shape.

My favorite part of the paper is that it attacks the big problem of optimal construction with reconfigurable robots, shows a non-trivial solution that builds at O(2^(2N)) more voxels per timestep, and relates the insights gained to biology. It is wonderful how complete the paper is, from including weight and cost of all the robot materials, the careful construction and algorithms for the robots, to the beautiful 3 videos. "

The paper has been edited and improved. It is both timely and well executed, so it should be appreciated by the robotics community.

We really appreciate the reviewer's comment. This is a very concise summary of the paper and our contribution.

"BIG ISSUES" In order of importance

no big issues. The paper was revised.

"MINOR EDITS"

The text in Build Sequence Tree in Fig. 4 is still a bitmap, not a vector

Fig. S4, B and C the plots (but not the legends) are still bitmaps, not vector graphics

Thank you for noticing that. We changed Fig. 4C to be in vector format as well as Fig. S4, B and C.

"Concerning the point raised about the detachment force, since the algorithm presented is only concerned with construction and not reconfiguration, the only two instances where the blocks need to be detached are when they are picked up from the pickup stations or when they are detached from the robot itself (the gripper)." --> but consider the large robot in Fig. 3, which is shown picking up a 4x4x4 voxel cube. This cube was presumably constructed by the smaller robots, so it would need to be picked up.

As mentioned in the previous reply, if they are constructed by the smaller robots (Like in Movie S3 – "Serial vs Hierarchical Construction" or "Construction and Path Planning Simulation") the intermediate blocks will be constructed in a designated area near the pickup stations. Hence the magnets of the substrate in these areas could be designed to be much weaker for it to be easier to be picked up by the larger robot, or with a special detachment mechanism. We think explaining this part in the main text might confuse the readers, and emphasize a discussion about magnets' scaling, which we mentioned in the discussion were only used for a demonstrative purpose and will not be optimum for scaling. Joints with improved stiffness and strength will need to be used.

Line 13: "Teams of wheeled robot arms can collectively 3D print a single object larger than their individual workspace." The workspace for a single wheeled robot is unbounded. Perhaps this should be "Teams of wheeled robot arms can collectively 3D print a single object larger than the workspace for any individual arm." Is there something that a team can do that a individual cannot in this example?

This has been edited. The reviewer is correct, the reference is an example of parallelization rather than an example of "something that a team can do that an individual cannot".

Line 131, please define L2.5. In figure 4, L means 'layer', but in 131-133 it seems to refer to an exponent term. Please also define L2 and L3.

This has been corrected. L was confusing, what we meant was *H* (size) and scaling factor of 2 or 3.

In Fig. 6, what does X% resolution mean? how is 0% a cube, and 90% a more complicated step pyramid? Presumably there is a simple equation being used here.

We thank the reviewer for the comment we added an explanation for the resolution in main text in line 204-205.

"TINY EDITS" (e.g. typos)

Abstract: it seems awkward to describe swarm + object construction as "compile a shape". Is there a better way, e.g. "compose building components into both a swarm and the desired object"?

This has been adjusted.

24: ", where the robots and the material they build are designed simultaneously and hence indistinguishable." -> ", where the robots and the material they build with are designed simultaneously and hence are indistinguishable." -- but is 'designed' the correct word here? Should this be "are designed to be indistinguishable.", since the purpose was to make the robot parts and building parts out of the same components?

This has been corrected as well.

Line 51, 52, x,by, z should be in math font

Corrected.

Line 57, N should be in math font

Corrected.

Fig. 1, A 'N' and 'H' should be in math font

Corrected.

Lines 63-66 are one long sentence. Please split this paragraph into several sentences.

Corrected.

Sentence 67 is awkward. 'but larger robots' -> but can also build larger robots'

Corrected.

Line 68 is also awkward. Perhaps remove word 'although' and clean this up.

Corrected.

Line 70, should 'N' be 'H'? Alternately, should lines 68-69 use 'N' instead?

In that instance N=H so we edited it for it to be clearer.

Line 71, 'N' in math font

Corrected.

Line 77, 'E' in math font

Corrected.

Line 78, swap order of 'we were' to 'were we'

Corrected.

Lines 97 and 100, 102 please add spaces between number and units.

Corrected.

Caption Fig. 3, 'H' in math mode

Corrected.

Line 143 "A task entails picking up a building block from the pickup station in form of a list of high-level steps instructions towards the drop-off location" -> "A task entails picking up a building block from the pickup station in the form of a list of high-level step instructions towards the drop-off location"

Corrected.

222 please use \cdot rather than '.' for Nm unit.

Corrected.

51, 362, 382, 383, 384, 386, 388, 389 (and in eq S2) please use \cdot rather than '' for multiplication*

Thanks for pointing this out. We have corrected it.

VIDEO:

these are great!

We thank the reviewer a lot for this comment.

Reviewer #2 (Remarks to the Author):

The authors provided clarifications for all concerns I raised previously. However, I could not always find a place in the manuscript for some of the mentioned clarifications.

1) I found in the rebuttal the sentence

"Even though [...] we introduced an new algorithm, a new path planning and task allocation strategy for recursive and hierarchical assembly, as well as novel scalable shape compiler for scalable hierarchical assembly.",

but I could not find such a clear statement of novelty with respect to the previous work in the manuscript. I would have expected to find such a statement in the last paragraph of the introduction for example.

We thank the reviewer for this comment, we have indeed added a sentence at the end of the last paragraph for the introduction to clarify this.

2) About the "aspects of structural integrity, and mechanical subsystem actuation/motion feasibility", I could not find the lines in the manuscript with the respective clarifications.

We added the clarification for the structural integrity in line 171-172. As for the mechanical subsystem actuation/motion feasibility, we added a sentence in line 224-227 with more clarification.

3) The sentence "I would suggest the authors to describe more formally the addressed problems in the introduction and directly motivate the significance for their solution." was not referring to a clarification to the top-level goal of the study, but to the technical problems faced instead -those that led to the novelties the authors mention in "1)" above-.

We added a sentence explaining the technical problems faced that led to the novelties mentioned in 1) (above). This can be found in the introduction in line 20-21.

4) I could not find a "section 4" in the supplementary materials for the assumptions. I can find a "Figure S4" mentioning some assumptions followed by text (is this section 4?). Anyhow, the fact that the Authors assume not to have actuation and power limits in the proposed algorithms should be mentioned in the paper.

We apologize for the confusion. We indeed meant the text following Figure S4 which explains in detail the assumptions used (edited in the paper). It was explained there that we have speed constraints for the larger robots, but we re-emphasized that in line 158-159 (caused by actuation limits). As for the power limits, we added a sentence in line 164 saying that the pickup stations could function as charging stations.

These limits can be changed in the algorithm if needed as they are macro variables. Plans for scaling and addressing limits on the hardware side are explained in the Discussion section in line 232-234.

5) I would find it to be clearer if the algorithms that constitute the main novelty of the paper were presented with pseudocode and a description of said pseudocode. Anyhow, I also respect other reviewers' opinion as well as Author's preference. Thus, feel free to keep the current presentation of the algorithms if that's your preference. However, it would be laudable if the Authors could share the code and data related to the main results as supplementary material or provide a link to a repository.

We respect the reviewer's opinion and we provided a link to the code's repository under Code Availability.

Reviewer #3 (Remarks to the Author):

The authors correctly answered all questions and doubts from the reviewer

We appreciate the reviewer's feedback. Thank you.

REVIEWERS' COMMENTS:

Reviewer #1 (Remarks to the Author):

This is great -- I'm really looking forward to sharing with students.
Typo: line 203, missing word 'to': subdivisions where the final voxelized structure is identical the input mesh)) -> subdivisions where the final voxelized structure is identical to the input mesh))

Fig. 3 right, I wish the 'R' were in the same math font as the caption and text

Reviewer #2 (Remarks to the Author):

The Authors provided clarifications considering my comments. I have no further comments.

Reviewer #3 (Remarks to the Author):

The authors correctly answered all questions and doubts from the reviewer

COMMSENG-22-0033B: Response to Reviews

Reviewers' comments:

We thank the reviewers very much for their time and feedback.

Reviewer #1 (Remarks to the Author):

This is great -- I'm really looking forward to sharing with students.

Thank you very much!

Typo: line 203, missing word 'to': subdivisions where the final voxelized structure is identical the input mesh)) -> subdivisions where the final voxelized structure is identical to the input mesh))

Done.

Fig. 3 right, I wish the 'R' were in the same math font as the caption and text

Done.

Reviewer #2 (Remarks to the Author):

The Authors provided clarifications considering my comments. I have no further comments.

We appreciate the reviewer's feedback. Thank you.

Reviewer #3 (Remarks to the Author):

The authors correctly answered all questions and doubts from the reviewer

We appreciate the reviewer's feedback. Thank you.
